# Effects of moderate thermal anomalies on *Acropora* corals around Sesoko Island, Okinawa

**DOI:** 10.1371/journal.pone.0210795

**Published:** 2019-01-30

**Authors:** Tanya Singh, Mariko Iijima, Ko Yasumoto, Kazuhiko Sakai

**Affiliations:** 1 Graduate School of Engineering and Science, University of the Ryukyus, Nishihara, Okinawa, Japan; 2 Kitasato University School of Marine Biosciences, Sagamihara, Kanagawa, Japan; 3 Sesoko Station, Tropical Biosphere Research Center, University of the Ryukyus, Motobu, Okinawa, Japan; Living Oceans Foundation, TAIWAN

## Abstract

Over the past several decades, coral reef ecosystems have experienced recurring bleaching events. These events were predominantly caused by thermal anomalies, which vary widely in terms of severity and spatio-temporal distribution. *Acropora* corals, highly prominent contributors to the structural complexity of Pacific coral reefs, are sensitive to thermal stress. Response of *Acropora* corals to extremely high temperature has been well documented. However, studies on the effects of moderately high temperature on *Acropora* corals are limited. In the summer of 2016, a moderate coral bleaching event due to moderately high temperature was observed around Sesoko Island, Okinawa, Japan. The objective of this study was to examine thermal tolerance patterns of *Acropora* corals, across reefs with low to moderate thermal exposure (degree heating weeks ~2–5°C week). Field surveys on permanent plots were conducted from October 2015 to April 2017 to compare the population dynamics of adult *Acropora* corals 6 months before and after the bleaching events around Sesoko Island. Variability in thermal stress response was driven primarily by the degree of thermal stress. Wave action and turbidity may have mediated the thermal stress. Tabular and digitate coral morphologies were the most tolerant and susceptible to thermal stress, respectively. Growth inhibition after bleaching was more pronounced in the larger digitate and corymbose coral morphologies. This study indicates that *Acropora* populations around Sesoko Island can tolerate short-term, moderate thermal challenges.

## Introduction

Coral reef ecosystems worldwide are being challenged by increasing global and local anthropogenic stress. Stressors can affect individual performance, community species composition, and consequently, ecosystem function [1–4]. Coral bleaching is caused by the collapse of the mutualistic relationship between host corals and their symbiotic algae and it is a major threat to the health and survival of coral reefs. Bleaching occurs mainly in response to rising average sea surface temperature (SST) with strong irradiance [5–8]. Bleached corals are physiologically stressed and this affects growth and mortality in coral populations [9,10]. Consequently, coral bleaching may transform the structure and functional diversity of coral communities [11].

In addition to its immediate effects, bleaching may also have long-term effects on corals after temperatures have returned to normal. Omori et al. [12] reported that the fertilization rates of *Acropora* corals decreased by ~50% in 1999 following the 1998 mass bleaching event on Aka Island in the Ryukyus. The bleaching event may have reduced sperm motility. Ward et al. [13] also reported zero reproductive output of both bleached and recovered corals on Heron Island, Great Barrier Reef (GBR), after the 1998 bleaching event. Muko et al. [14] found that the coral recruitment rates after bleaching events were lower than those before on Iriomote Island in the Ryukyus. The prolonged effects of bleaching on coral growth are inconsistent. Some studies reported reduced coral growth after a thermal anomaly [15], while others indicated that the growth rates of surviving colonies were unaffected by bleaching [14]. High coral mortality rates are typical immediately following a severe bleaching event [10,16–18]. In contrast, extended or prolonged coral mortality 6–8 months after a bleaching event was observed at the GBR [11]. Understanding the risks and mechanisms of the long-term prolonged effects of bleaching on coral populations will help us to predict future shifts in coral community health and functioning.

Many coral reefs globally, including those in the Ryukyu Islands of Japan, experienced a severe bleaching event in 1998 [19]. At Sesoko Island, the corals were severely affected by this bleaching event [20]; up to 85% of the hard and soft coral cover was lost. The massive hard coral morphologies, like *Porites*, were the survivors, whereas the branching hard coral morphologies such as *Acropora* and the pocilloporids were more severely affected [20]. Similar morphology-specific bleaching susceptibility has been reported for other coral reefs [21,22]. In 2016, severe bleaching events (>60% corals bleached) occurred on many reefs worldwide [11], including the Ryukyu Islands [23]. Nevertheless, bleaching-induced mortality of *Acropora* corals (thermally vulnerable taxa in the 1998 bleaching event) [20] was lower in 2016 than it was in 1998 on the Sesoko Island reef; all *Acropora* colonies larger than 10 cm in diameter died during the 1998 bleaching event [20]. In this study, we observed the effects of moderate thermal stress on branching *Acropora* corals. This genus dominates in many reefs in the Ryukyu Islands. Its member species show high morphological diversity and provide a three-dimensional habitat for other reef organisms.

The life history and morphological traits of corals may determine their thermal stress tolerance. It has been postulated that compared to fast-growing branching species, slow-growing massive species have higher thermal tolerance [11,22,24–26]. Morphological traits have been assessed at the polyp and colony levels. The thicker polyp tissue of massive corals compared to that of branching corals provides shade for the symbiotic algae within the coral cells via polyp tissue retraction. This feature may, in part, account for the relatively higher thermal tolerance of massive corals [19,20,27]. At the colony level, interspecific variations like encrusting vs. branched colonies and intraspecific variation such as small vs. large colonies have been discussed in terms of their relative differences in mass flux rate. High mass flux rates are associated with the efficient removal of oxidative metabolites by diffusion [20,28].

*Acropora* corals most commonly have a branching colony morphology. However the genus is morphologically diverse and includes corymbose, digitate, tabular, and arborescent forms [29]. In this study, we excluded tissue thickness from the discussion of thermal tolerance differences because all *Acropora* corals have similar tissue thickness [20]. We therefore evaluated the effects of colony morphology and growth on thermal stress tolerance among various *Acropora* species. We also compared the effects of thermal stress after bleaching events on the growth of colonies of different sizes within the same species.

Seawater temperature, cloud cover, wind force, seawater turbidity, reef microhabitat structure (such as coral overhang and crevices), water flow, wave action, and depth may all reduce thermal stress and cause coral bleaching response heterogeneity on a small spatial scale (≤tens of km) [21,30–33]. Degree heating weeks (DHW) is an index of accumulated heat exposure over 12 weeks [34–37], and therefore it considers both intensity and duration of thermal exposure. DHW of 4°C-week usually results in significant bleaching and 8°C-week results in critical, wide-spread bleaching and significant mortality [35]. Following these criteria, DHW between 4–8°C-week are defined as representing a moderate thermal anomaly in this study. DHW has been widely used to quantify bleaching thresholds and to asses thermal stress variability on a large spatial scale (≥hundreds of km) [38–40]. Small-scale thermal disparity and consequent differential bleaching responses have been observed [33,41]. Nevertheless, to the best of our knowledge, no studies have used DHW to determine thermal exposure variability on a small spatial scale.

In the present study, we explored the prolonged or extended effects of bleaching in *Acropora* corals. We compared *Acropora* coral population dynamics before and after a moderate thermal anomaly in different environmental regimes and examined whether (1) bleaching prevalence is driven primarily by the degree of thermal exposure, (2) *Acropora* demographic rates recover after the temperature returns to normal, and (3) *Acropora* colony morphological traits determine inter and intraspecific differences in thermal stress tolerance.

## Methods

### Ethics statement

No permission was required to survey coral reefs in the study area. Only digital images of corals were collected, no fauna or flora were collected or manipulated in this study.

### Study area

**Fig 1:**
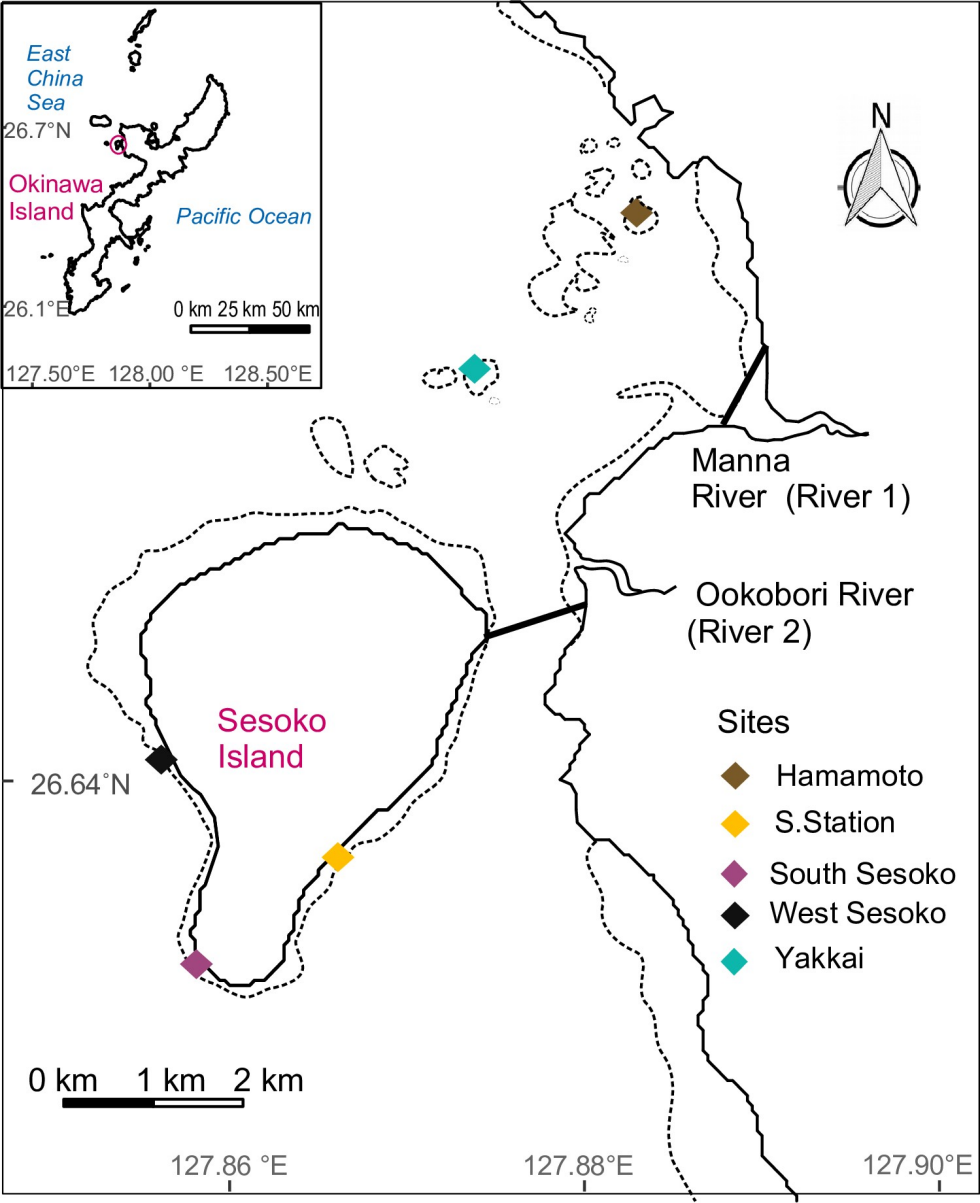
Map of Sesoko Island showing study area. Differently colored rhombuses represent study sites. Dotted lines represent reef edges.

This study was conducted around Sesoko Island, Japan (26.646°N, 127.86°E, Fig 1) between October 2015 and April 2017. Field surveys were conducted four times, in October 2015, April 2016, October 2016, and April 2017. Sesoko Island is situated near the northern Okinawa Island in the Ryukyu Islands. Five sites around Sesoko Island were selected: Hamamoto (26.671672°N, 127.882922°E), Yakkai (26.663028°N, 127.873797°E), Sesoko Station (26.63598°N, 127.8661046°E), Sesoko South (26.63005°N, 127.858125°E), and Sesoko West (26.641375°N, 127.856147°E).

The influence of variability in the degree of thermal anomalies and other environmental parameters on bleaching on a small spatial scale was evaluated (~5 km). The study area had varying degrees of thermal exposure, anthropogenic stress/turbidity, and wave exposure (Table 1). Seawater temperature in the reefs was measured continuously, but anthropogenic stress and wave exposure were assessed qualitatively. Two rivers were situated near our study sites. Nitrate (NO_3_) concentration in river 1 and 2 ranged from 0.7 ± 0.1 (mean ± SD) to 70.7 ± 27.9 μM and 2.8 ± 1.8 to 56.4 ± 1.7 μM, respectively (further details in S1 File). Suspended particulate matter in river 1 from 1976 to 1996 was 19.2 ± 22.7 mg/L [42]. Additionally, considerable pesticides such as diuron (<0.7 μg kg^−1^), irgarol (<0.016 μg kg^−1^), and chlorpyrifos (<0.41 μg kg^−1^) have been previously detected in sediments of river 1 [43]. Hence, the distance from the two rivers to the study area (Fig 1) is provided as a proxy for anthropogenic stress.

**Table 1:** Environment variability at study sites around Sesoko Island.

			Proxy for Anthropogenic Stress				
Site	Depth -High tide	Distance from the shore	Distance—river 1	Distance—river 2	Turbidity	Wave Exposure	Thermal exposure
Hamamoto	2 m	0.59 km	1.33 km	2.23 km	High	Low	Intermediate
Yakkai	2 m	1.06 km	1.53 km	1.29 km	High	Low	Intermediate
Sesoko Station	2 m	0.08 km	3.88 km	2.19 km	Intermediate	Intermediate	High
South Sesoko	3 m	0.07 km	5.26 km	3.58 km	Low	High	Intermediate
West Sesoko	4 m	0.08 km	4.66 km	3.82 km	Low	Moderate	Low

### Temperature and Degree Heating Week (DHW) measurement

One temperature logger was deployed at each study site from April 2016 to April 2017. From April 2016 till October 2016 HOBO water temp pro v2 (Onset, Cape Cod, MA, USA) was deployed, and from October 2016 to April 2017 HOBO pendant loggers (Onset, Cape Cod, MA, USA) were deployed. Hourly temperature data were collected from all sites. Weekly mean temperatures per site were calculated from the hourly temperature data set. Then weekly hotspots per site were calculated by subtracting the weekly mean temperature from the maximum monthly mean (28.9°C) temperature of Okinawa, which was obtained from National Oceanic and Atmospheric Administration (NOAA) coral reef watch website [38]. DHWs were calculated by summating the average weekly temperatures of hotspots (≥1°C) of the previous 12 weeks [35–37].

### Survey design

A hierarchical survey design was followed. At each site, two sub-sites ~30–60 m apart were selected. At each sub-site, two permanent 2 m × 2 m quadrats ~10 m apart were established. Each quadrat was divided into sixteen 50 cm × 50 cm sub-quadrats. A picture of each sub-quadrat was taken perpendicular to the quadrat plane using a digital camera (Canon S95 in Canon WP-DC38 waterproof case; Canon Inc., Tokyo, Japan) fitted with a wide-angle lens (INON UWL-H100 28M67, 0.60 mm; INON Inc., Japan). Close-up images were taken of all small colonies not detectable in the 50 cm × 50 cm images. Five-centimeter scales were placed on or near all *Acropora* colonies for precise image calibration.

### Adult *Acropora* size and void ratio

Projected area, branch spacing (S; n≈10–20/colony), and branch diameter (d; n≈10–20/colony) were determined from the digital images with ImageJ v.1.51a [44]. Calibrations were made using the 5-cm scales. All *Acropora* colonies >5 cm in diameter were assumed to be adults. These were classified into four morphological groups: arborescent, tabular, corymbose, and digitate (S1 Fig). Arborescent colony boundaries could not be determined; therefore, the growth rates of this colony morphology were not measured, and they were removed from the analysis. To monitor colony growth, a projected colony area was measured at all sites in October 2015, April and October 2016, and April 2017. Void Ratio (VR) is defined as the porosity of a coral, i.e., the volume of open space in a coral relative to closed space[45]. Coral morphologies with higher void ratios have been shown to have higher mass flux rates [45], while higher mass flux rates have been attributed to bleaching or thermal tolerance of corals [20]. VR was calculated to test if different *Acropora* morphologies had similar VR and thermal susceptibility patterns in this study. The following formula was used [45]:

(VR = (average S + average d)/average d). VR was measured only at Sesoko Station in March 2018 because spatial variation in the VR of a species is expected to be negligible [45]. VR was measured for nine colonies of digitate and four colonies of each corymbose and tabular morphologies.

### Bleaching prevalence

Bleaching prevalence was visually assessed during the October 2016 survey. Here, bleaching is defined as when colonies exhibited a pale color and could be partial bleaching or complete bleaching. The bleaching prevalence for all morphologies at each site was expressed as a percentage of the total number of bleached colonies.

### Statistical analysis

Semi-annual growth and partial mortality were calculated for two time periods. Time period one (t1) was from October 2015 to April 2016. Time period two (t2) was from October 2016 to April 2017. SST was higher than normal in the summer of 2016 (S2 Fig) and corals including *Acropora* spp. bleached (see results; [23]); therefore, t1 and t2 were defined as before and after the bleaching period, respectively. Only adult colonies first observed in the October 2015 survey and still alive in all subsequent surveys (433 colonies) were included in the growth analysis. Growth was expressed as the change in colony size within the projected area. Herein, the terms positive, zero, and negative (partial mortality) refer to growth.

The growth dataset was also split according to colony morphology (tabular, digitate, and corymbose). Sites with <5 colonies per morphology per time period were removed from the analysis. Thus, digitate and tabular morphologies were not included in the analysis at Hamamoto and Yakkai, respectively. Outlier colonies for growth decrements were assessed by Cleveland dot charts (S3 Fig) [46] and subsequently one tabular colony (−2986 cm^2^/6 months), and two corymbose (≤−158 cm^2^/6 months) and digitate colonies (≤−732 cm^2^/6 months) with the highest growth decrements were also removed from the analysis. Colony size was log-transformed in the analyses. A linear mixed regression model was developed using time period, site, initial size (as a covariate), and their interactions as fixed effects, and sub-sites and quadrats as random effects. As random effects did not improve the model fit nor had significant effect, they were removed from the model. Residual plots, however, suggested heterogeneity across all explanatory variables. We attempted to resolve this issue by following alternative methods.

Growth data were transformed with the following formula: sign(value) × (absolute (value))^1/2^. This formula is appropriate for datasets with both negative and positive values. Transformed growth data were then modelled using simple linear regression (Ordinal Least square /OLS) and Partial Least Square (PLS) regression. PLS regression is a robust technique, which is useful when several explanatory variables with multicollinearity are present. We also modelled untransformed growth data by Generalized Least Square (GLS) regression. In this method a variance structure for variables showing heterogenous patterns can be included in the model as weight. Parsimonious GLS models were chosen by first selecting a variance structure and then an explanatory variable combination. Models with all explanatory variables and different combinations of variance structure were fitted. The best variance structure was selected by comparing Akaike Information Criteria (AIC) values of all GLS models. Variance in the Ln-size was specified by a varPower function for all morphologies. Variances in both site and time were specified for tabular colonies by a varIdent function. Only time and site were specified for digitate and corymbose colonies, respectively. The best explanatory variable combination was selected by stepwise backward variable elimination, to find a model with the lowest AIC values for both OLS and GLS regression. If the difference in the AIC of two models was less than two, then the model with the least number of parameters was selected as the best fit model. Best fit PLS regression models were constructed based on Variable Importance (VIP) and coefficient values of explanatory variables. Best-fit GLS plots showed (S4–S6 Figs) homogenous residual patterns, whereas best-fit OLS and PLS models showed heterogenous residual patterns. OLS and PLS regression models were therefore discarded. To test for the significance of each variable, marginal ANOVA was performed on the best-fit GLS growth models. Further post-hoc tests by pairwise comparisons of least square means with Tukey adjustments were performed, to test for significance within each interaction term. Normal distribution of the best-fit GLS model’s residuals were observed.

Whole colony mortality rates were calculated for t1 and t2. Mortality was also calculated immediately after bleaching from April 2016 to October 2016 (tbl). There were 560 colonies examined in t1, 522 in tbl, and 476 in t2. Dead colonies were assigned a value of 1 and living colonies were given a value of 0. Various binomial Generalized Linear Model (GLM) regressions were applied with a logit link function. First, temporal variations were tested at different sites (all morphological categories were pooled by site). Significant temporal variations were observed only at Sesoko Station; therefore, the three best-fit binomial GLM models were developed for each time period. The following explanatory variables were included in these models: site, time, Ln-size, colony morphology, and their interactions. Stepwise backward variable elimination was carried out, to find a model with the lowest AIC values. χ^2^ tests were carried out on GLM models to test for the significance of each variable. Post-hoc tests by pairwise least square means comparison with Tukey adjustment was performed to test for significance within the interaction terms.

One-way ANOVA and pairwise comparison of least square means with Tukey adjustment methods were used to test if VR differed among morphology groups. GLS and GLM model specification and selection protocols were based on Zuur et al. [46] and Zuur et al. [47], respectively. PLS analysis was carried out using JMP Pro 13 software (SAS Institute Inc., Cary, NC). All other analyses were performed using R v. 3.3.3 [48] and the following packages: nlme [49], lmertest [50], stat, car [51], lsmeans [52], multcomp [53], AICcmodavg [54], and ggplot2 [55].

## Results

### Species composition

Corals with corymbose morphologies (N = 146) comprised *Acropora digitifera*, *A*. *nasuta*, *A*. *latistella*, *A*. *cerealis*, *A*. *nana*, and *A*. *valida*. Those with digitate morphologies (N = 328) comprised *A*. *gemmifera*, *A*. *monticulosa*, *A*. *humilis*, *a few colonies of A*. *digitifera*, *and A*. *nasuta*. Those with tabular colonies (N = 69) comprised the *A*. *hyacinthus* species complex [56] and *A*. *cytherea*.

### Degree heating week (DHW)

**Fig 2:**
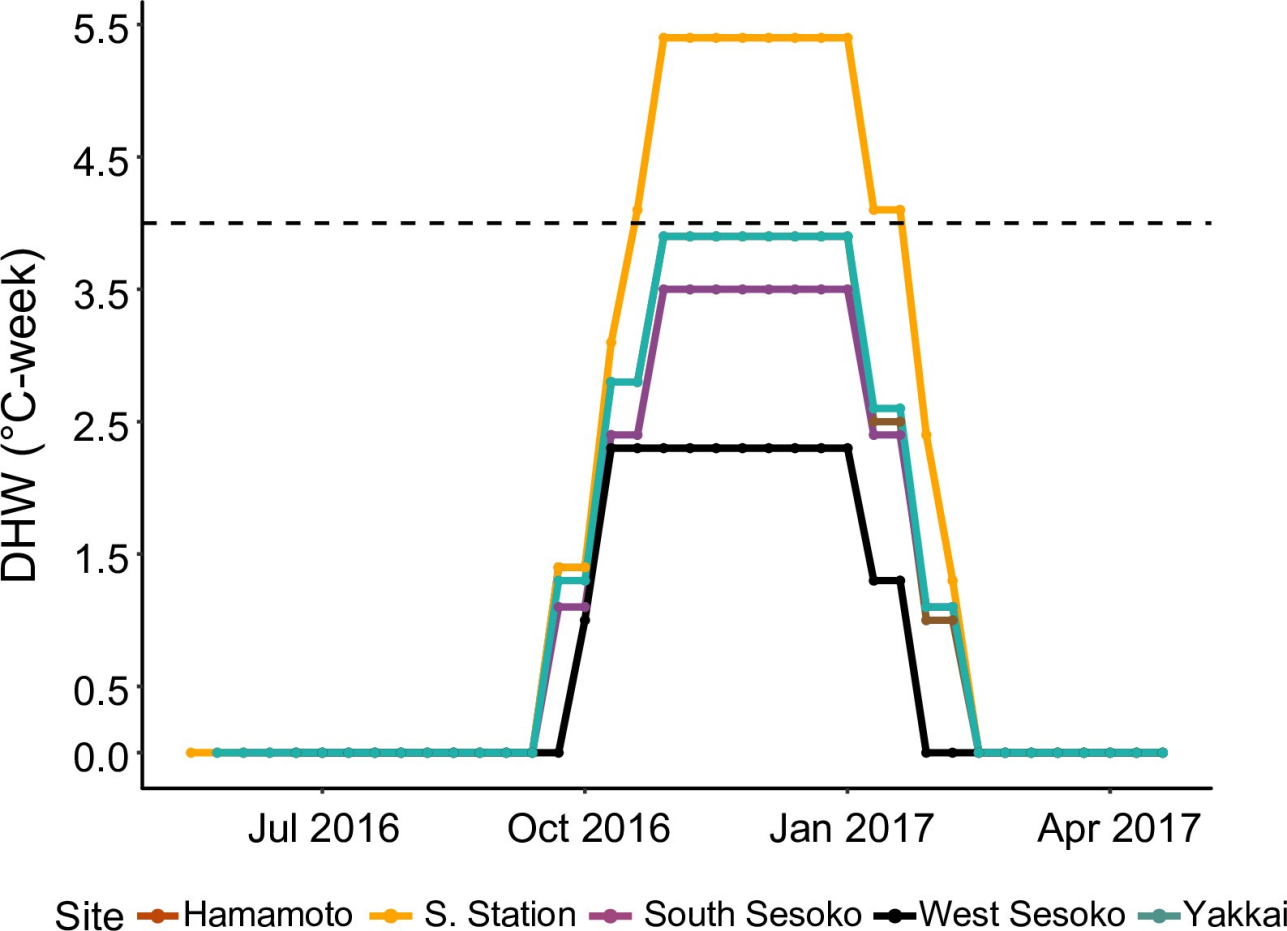
Spatio-temporal variation in DHW around Sesoko Island. Dashed line at 4°C-weeks indicates the significant bleaching level.

DHW reached above the significant bleaching level of 4°C-weeks only at Sesoko Station (1.4 to 5.4°C-weeks, Fig 2). Whereas, the DHW at Hamamoto and Yakkai reached just below the significant bleaching level (1 to 3.9°C-weeks, Fig 2). DHW at South Sesoko and West Sesoko ranged from 1.1 to 3.5°C-weeks and 1 to 2.3°C-weeks, respectively (Fig 2). The duration of ≥1°C-weeks DHW was 13 weeks at West Sesoko and 16 weeks at all other sites. In this study, Sesoko Station experienced a moderate thermal anomaly, West Sesoko experienced a low thermal anomaly, and Hamamoto, Yakkai, and South Sesoko experienced an intermediate thermal anomaly.

### Spatial variation in the degree of bleaching

**Fig 3:**
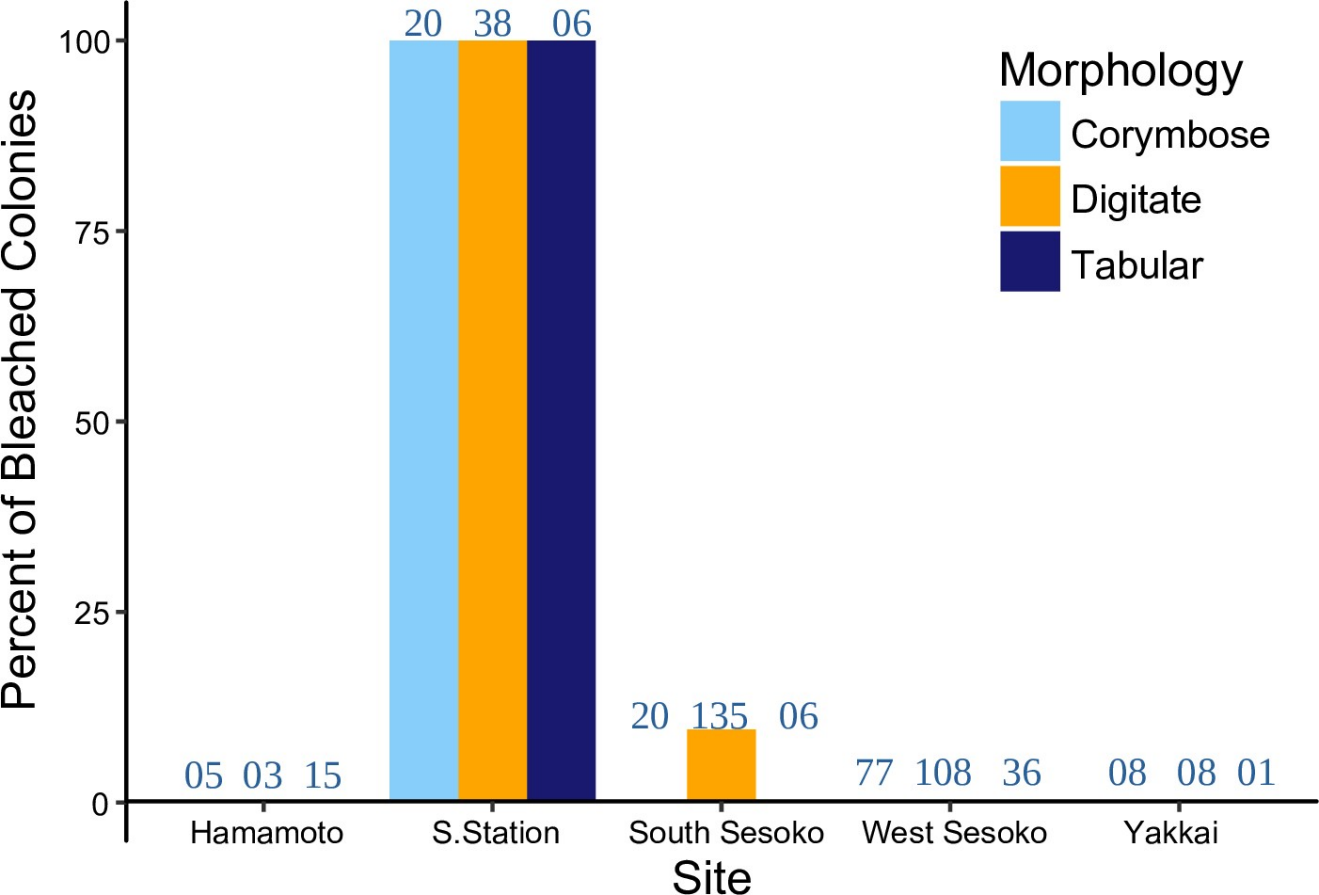
Spatial variation in bleaching prevalence (percent of bleached colonies) of the different *Acropora* morphologies around Sesoko Island in October 2016. Sample numbers per group are in blue text.

The degree of bleaching of *Acropora* corals was variable among sites. In October 2016, bleaching was observed mainly at Sesoko Station (Fig 3). All colonies of all morphologies were bleached at Sesoko Station. At South Sesoko, 9.6% of the digitate colonies were bleached but the tabular and corymbose colonies were not affected. None of the colonies were bleached at West Sesoko, Hamamoto, or Yakkai.

### Spatio-temporal variation in growth

Seventeen possible GLS regression models were fitted to analyze the spatio-temporal variation in the growth of each *Acropora* morphology. Models with the lowest AIC values were selected as the best-fit models (Table 2). Pseudo R^2^ values for the best-fit models describing the digitate, corymbose, and tabular growth rates were 30.6%, 45.8%, and 75.7%, respectively. The results of the ANOVA carried out on the best-fit GLS models indicated that all morphologies showed site-specific growth and Ln-size relationship (Initial size*Site—*p* <*0*.*001* for all morphologies; Table 3). The decrease in growth of the digitate colonies was both site and initial size-specific (digitate: Time*Site—*p* <*0*.*001*; Time*Initial size—*p* = *0*.*001*; Table 3), whereas it was only size-specific for corymbose colonies (Time*Initial size—*p* = *0*.*005*; Table 3).

**Table 2:** AIC values of different GLS models of growth for each morphology. Best-fit models with lowest AIC values for each morphology are bolded and underlined. “*” Denotes an interaction between two variables.

Model No.	Model Parameters	Digitate AIC Values	Corymbose AIC Values	Tabular AIC values
**M full**	Initial size + Site + Time + Initial size*Site + Initial size*Time + Site*Time	**5039.2**	2276.9	1393.2
M1	Initial size + Site + Time + Initial size*Time + Site*Time	5052.0	2297.1	1403.0
**M2**	Initial size + Site + Time + Initial size*Site + Site*Time	5048.6	2283.8	**1392.0**
**M3**	Initial size + Site + Time + Initial size*Site + Initial size*Time	5059.9	**2276.3**	1396.6
M4	Initial size + Site + Time + Site*Time	5068.3	2303.0	1401.8
M5	Initial size + Site + Time + Initial size*Site	5065.1	2282.2	1394.8
M6	Initial size + Site + Time + Initial size*Time	5076.1	2289.9	1404.1
M7	Site + Time + Site*Time	5067.7	2317.1	1450.4
M8	Initial size + Site + Initial size*Site	5068.3	2282.7	1393.2
M9	Initial size + Time + Initial size*Time	5132.0	2294.9	1408.2
M10	Initial size + Site + Time	5085.6	2297.0	1402.5
M11	Initial size + Time	5136.5	2300.9	1407.4
M12	Initial size + Site	5084.8	2295.9	1400.9
M13	Site + Time	5083.8	2310.7	1448.6
M14	Time	5137.7	2335.3	1452.5
M15	Site	5083.3	2315.6	1447.8
M16	Initial size	5139.7	2300.1	1405.7

**Table 3:** *F* & *p* values of ANOVA tests carried out on best-fit GLS growth models. Gray cells indicate that these terms were not included in the best-fit model.

Model Parameters	Digitate		Corymbose		Tabular	
	*F* value	*p* value	*F* value	*p* value	*F* value	*p* value
Time	*0*.*38*	*0*.*540*	*6*.*32*	*0*.*013*	*2*.*50*	*0*.*116*
Site	*4*.*09*	*0*.*007*	*3*.*98*	*0*.*004*	*5*.*54*	***0*.*001***
Initial size	*1*.*47*	*0*.*226*	*10*.*31*	*0*.*002*	*22*.*99*	***<0*.*001***
Time*Site	*8*.*89*	***<0*.*001***			*2*.*58*	*0*.*057*
Site*Initial size	*6*.*21*	***<0*.*001***	*6*.*18*	***<0*.*001***	*6*.*46*	***0*.*001***
Time*Initial size	*11*.*34*	***0*.*001***	*8*.*24*	***0*.*005***		

### Site-specific decline in growth

**Fig 4:**
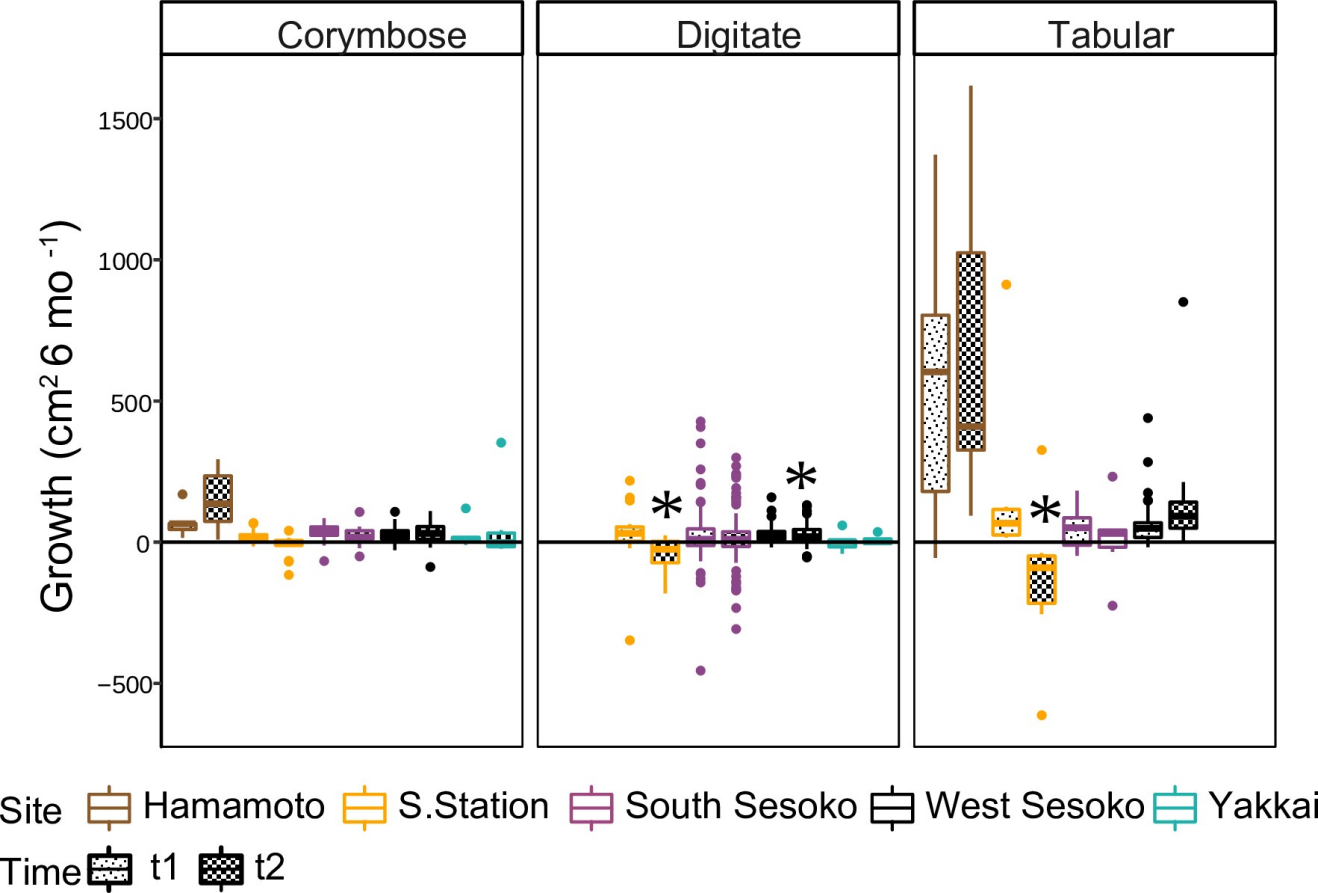
Spatio-temporal growth variation among *Acropora* morphologies at different study sites. Left and right bars of each pair represent growth in t1 and t2, respectively. Stars represent significant decrease of growth in t2.

The results of the ANOVA tests carried out on the best-fit models indicated that growth did not decrease significantly in the tabular colonies during t2 (Time*Site—*p* = *0*.*057;* Time—*p* = *0*.*116;* Table 3;Table A in S2 File). The post hoc tests on the Time*Site term, however, showed that growth of tabular colonies declined in t2 only at Sesoko Station (*p* = *0*.*039*; Table 4 and Fig 4). The post hoc tests for the digitate colonies showed that growth declined significantly at both Sesoko Station and West Sesoko in t2 (Sesoko Station *p* <*0*.*001*, West Sesoko *p* = *0*.*001*; Table 4 and Fig 4). The Site*Time interaction term was not present in the best-fit growth model of corymbose colonies.

**Table 4:** Post-Hoc tests for the Site*Time term used in the best-fit growth models of all morphologies. Least-square means of growth are compared between different site pairs. The results are averaged over the levels of Site. The level of significance was 0.05. Df = degrees of freedom; SE = standard error.

Morphology	Site	Time pairs	Estimate	Std. Error	Df	t-ratio	*p* value
Digitate	**Sesoko Station**	t1-t2	*50*.*71*	*8*.*53*	*479*	*5*.*95*	***<0*.*001***
	South Sesoko	t1-t2	*6*.*58*	*5*.*68*	*479*	*1*.*16*	*0*.*248*
	**West Sesoko**	t1-t2	*20*.*77*	*5*.*44*	*479*	*3*.*82*	***0*.*001***
	Yakkai	t1-t2	*15*.*00*	*10*.*38*	*479*	*1*.*45*	*0*.*149*
Corymbose	Site*Time interaction term was not included in the best-fit model						
Tabular	Hamamoto	t1-t2	*149*.*93*	*94*.*78*	*106*	*1*.*58*	*0*.*117*
	**Sesoko Station**	t1-t2	*92*.*86*	*44*.*39*	*106*	*2*.*09*	***0*.*039***
	South Sesoko	t1-t2	*71*.*42*	*62*.*52*	*106*	*1*.*14*	*0*.*256*
	West Sesoko	t1-t2	*1*.*33*	*5*.*78*	*106*	*0*.*23*	*0*.*820*

### Colony size-specific decline in growth

**Fig 5:**
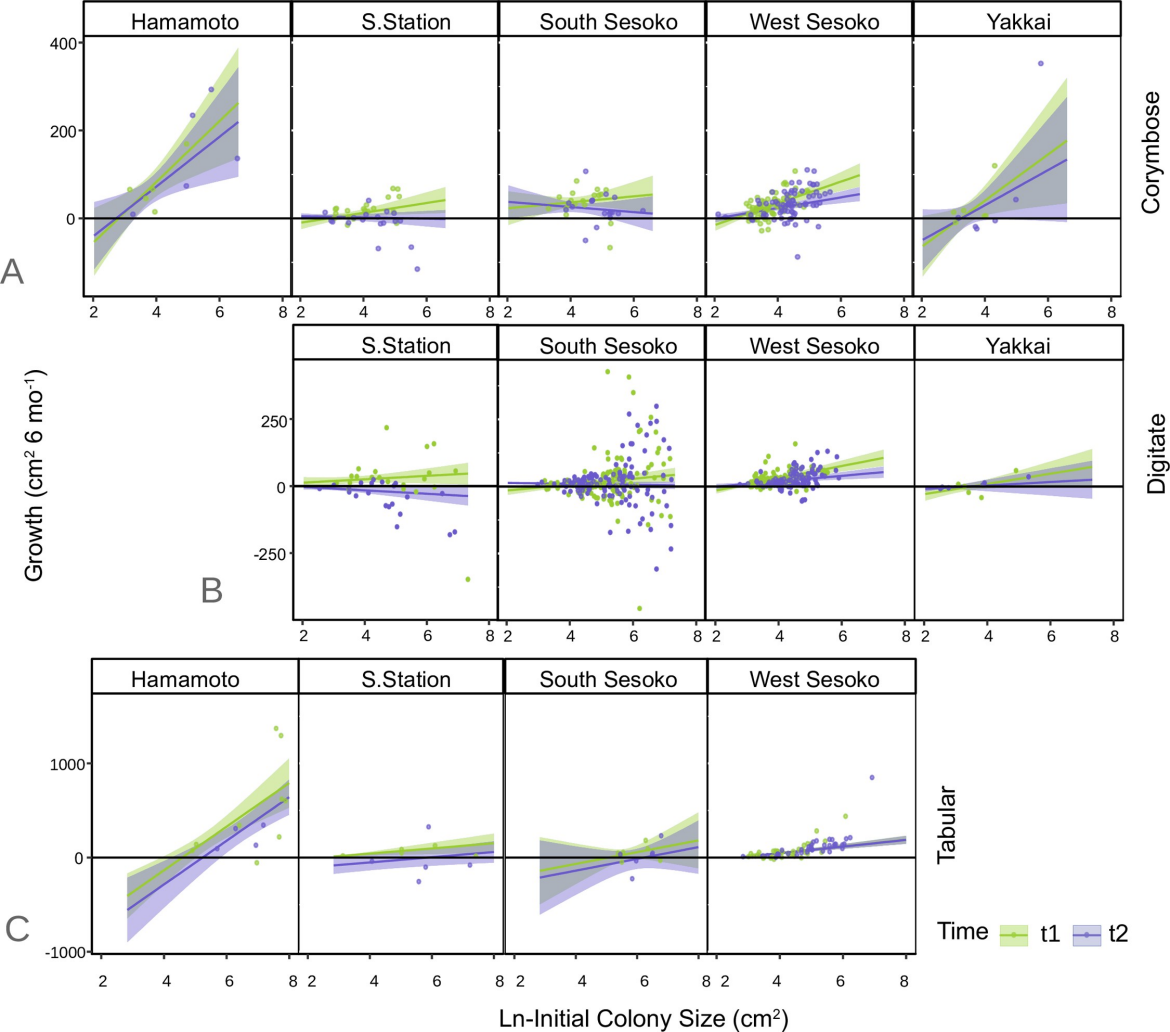
Initial size and growth relationship. Spatio-temporal variation in initial size and growth relationship of corymbose (A), digitate (B), and tabular (C) colonies. Line represents predicted slopes and 95% confidence intervals at different time periods.

For both corymbose and digitate colonies, the slopes of the regression between initial size and growth declined significantly in t2 (Time*Initial size *p* = *0*.*005* and *0*.*001* for corymbose and digitate respectively; Table 3 and Table A in S2 File and Fig 5A and 5B). At both Sesoko Station and South Sesoko, the positive correlations detected between initial size and growth in t1 became negative in t2. The size-specific growth decline was spatially variable (Fig 5). Overlapping confidence intervals at t1 and t2 for all colony morphologies at Hamamoto, South Sesoko, and Yakkai indicated that *Acropora* growth was less severely affected during t2 than it was at West Sesoko and Sesoko Station. At the latter two sites, the intervals did not overlap. At all sites, the slopes were parallel at t1 and t2 for the tabular colonies. In contrast, the slopes were not parallel at t1 and t2 for the corymbose and digitate colonies at all sites. Hamamoto and Yakkai were not included in the digitate and tabular morphology datasets respectively, due to low sample size. There were significant interactions between initial size and time for the digitate (*p* = *0*.*001;* Table 3) and corymbose (*p* = *0*.*005*; Table 3) morphologies. This interaction was excluded from the best model for tabular morphology because it increased the AIC and was not significant (*p* = *0*.*417*).

### Morphological variations in mortality after bleaching event

**Fig 6:**
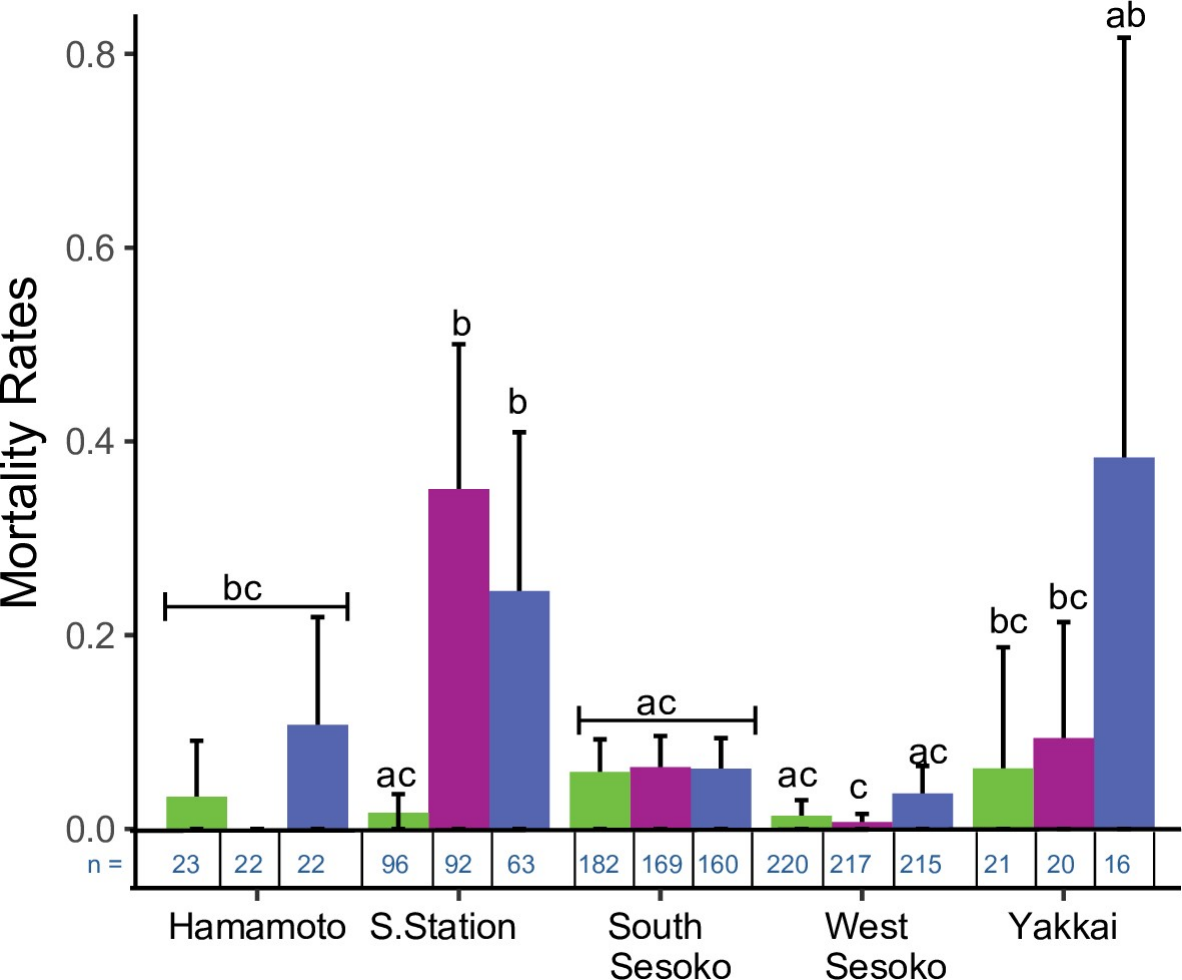
Spatial variations in mortality at different time periods. Letters represent significantly different groups. Error bars denote standard deviation. Sample size per treatment is below the graphs in blue text. t1: time period 1; tbl: 1 month after bleaching; t2: 6 months after bleaching. Different morphology categories are pooled by site.

Spatio-temporal variation in mortality rates was tested using a binomial GLM. χ^2^ tests on this GLM model showed that temporal variation in mortality rates was site specific (Time*Site *p* = *0*.*024*; Table 5 and Fig 6). It was significant at Sesoko Station, but not at the other sites (*p* ranged from *0*.*121–1*.*000*; Table 6 and Fig 6). Mortality rates increased significantly from <2% in t1 to 35% in tbl (*p* <0.001) and 24% in t2 (*p* = *0*.*001*) at Sesoko Station (Table B in S2 File). At Sesoko Station in t1 and tbl, null models were the best-fit models with the lowest AIC values (Table B in S2 File). Mortality rates at Sesoko Station were independent of initial size and morphology in t1 and tbl (Table B in S2 File). In t2, two models, one with only morphology and another with only morphology and initial size (Model no. 2 and 4; Table B in S2 File) as explanatory variables were the best-fit models. χ^2^ test of Model no. 4 showed that morphology had a significant effect (*p = 0*.*047*) while initial size (*p = 0*.*145*; Table C in S2 File) did not have a significant effect on mortality rates. In t2, mortality was observed in 41% (n = 37) digitate colonies, 5% (n = 20) corymbose colonies, and none of the tabular colonies (n = 6). At West Sesoko, 77% (n = 9) of colonies that died during t2 were also digitate.

**Table 5:** χ^2^ tests on spatio-temporal variability of GLM fitted mortality rates (all morphologies pooled).

Model Parameters	Mortality Rates	
	Pr (>Chisq)	Chisq
Time	*1*.*09*	*0*.*580*
Site	*6*.*47*	*0*.*170*
Time*Site	***17*.*61***	***0*.*024***

**Table 6:** Post-hoc tests to compare temporal variations in mortality rates at different sites. The least-square means of mortality rates are compared between different time periods. The results are on a logit scale. The level of significance was 0.05. SE = Standard Error.

Site	Time-pairs	odds.ratio	SE	z.ratio	p.value
Hamamoto	t1-t2	*0*.*29*	*0*.*34*	*-1*.*04*	*0*.*551*
	t1-tbl	*7*.*11E+05*	*3*.*64E+08*	*0*.*03*	*1*.*000*
	t2-tbl	*2*.*47E+06*	*1*.*26E+09*	*0*.*03*	*1*.*000*
**Sesoko Station**	t1-t2	*0*.*06*	*0*.*05*	*-3*.*60*	***0*.*001***
	t1-tbl	*0*.*05*	*0*.*03*	*-4*.*13*	***<0*.*001***
	t2-tbl	*0*.*73*	*0*.*27*	*-0*.*88*	*0*.*654*
South Sesoko	t1-t2	*0*.*97*	*0*.*44*	*-0*.*08*	*0*.*997*
	t1-tbl	*0*.*92*	*0*.*41*	*-0*.*18*	*0*.*982*
	t2-tbl	*0*.*96*	*0*.*43*	*-0*.*10*	*0*.*995*
west Sesoko	t1-t2	*0*.*32*	*0*.*21*	*-1*.*71*	*0*.*202*
	t1-tbl	*1*.*49*	*1*.*36*	*0*.*43*	*0*.*902*
	t2-tbl	*4*.*70*	*3*.*70*	*1*.*96*	*0*.*121*
Yakkai	t1-t2	*0*.*22*	*0*.*26*	*-1*.*27*	*0*.*415*
	t1-tbl	*0*.*28*	*0*.*34*	*-1*.*05*	*0*.*545*
	t2-tbl	*1*.*31*	*1*.*17*	*0*.*30*	*0*.*952*

### VRs

VR varied significantly with morphology (*p value = 0*.*004*, Table D in S2 File). Digitate colonies had lowest VR of 1.46 ± 0.18 (Table 7). Corymbose colonies had a significantly higher VR than digitate colonies (p = *0*.*004*, Table E in S2 File). VR of tabular colonies was similar to both corymbose (*p = 0*.*425*) and digitate colonies (*p value = 0*.*068*).

**Table 7:** Predicted mass transfer coefficients of different shapes in laminar/ turbulent flow (Patterson 1992) and void ratios calculated from close-up images of corresponding *Acropora* morphologies.

Geometric Shape	Corresponding Morphology	Mass Transfer	VR (this study)	Thermal sensitivity
Thickening cylinders (height ≤ width)	Digitate	0.31/0.36	1.46 ± 0.18	High
Thinning cylinders (height > width)	Corymbose	0.54/0.64	1.87 ± 0.20	Moderate
Flat/plate-like organisms	Tabular	1.25/1.1	1.72 ± 0.09	Low

### Discussion

The results of the present study indicate that the degree of coral bleaching may vary among reefs within a small spatial range, such as several kilometers, primarily owing to the relative differences in thermal exposure among reefs (Figs 2, 3 and S2). The maximum daily temperature and daily temperature fluctuations were significantly higher at Sesoko Station than at all other sites within a 5 km range (S2 Fig and S3 File), in addition the DHW at Sesoko Station was also highest (Fig 2). Compared to other shallow sites (Hamamoto and Yakkai) Sesoko Station was also closest to the shore (Table 1). This might explain the higher temperature regime at this site. Sesoko Station was the only site where all *Acropora* colonies were bleached irrespective of their morphology in the summer of 2016 (Fig 3). Bleaching-induced mortality and suppressed growth rates were observed for all morphologies following the bleaching event only at Sesoko Station. Relative to global bleaching events, Sesoko station was exposed to moderate thermal stress [4,23]. Moderate thermal anomalies are known to elicit a stress response in corals. For example, some sites at Florida Keys experiencing DHW ≤3°C-weeks resulted in a loss of 5% Shannon diversity [57]. Moderate temperature anomalies (+ 1.8°C) at Aka Island, Okinawa, Japan resulted in narrower size-class distribution of corymbose *Acropora* due to size specific mortality rates [58]. Our study additionally showed that, in the absence of a local environment filter, even mild to moderate thermal anomalies can result in prolonged effects such as depressed growth rates and increased mortality rates. Variations on a small spatial scale may have implications for the local conservation of coral reefs in the Anthropocene (sensu [4]). To preserve coral larval sources and sinks, corals located on reefs where the temperature is low, which are remote from local disturbances like crown of thorns starfish (COTS) predation and construction activity should be selected for the conservation.

Prolonged mortality was observed in the digitate colonies at Sesoko Station after the 2016 bleaching event even when temperature had already returned to normal. The mortality rates immediately after bleaching were both size- and morphology-independent at Sesoko Station. Six months after bleaching, mortality of the digitate colonies continued at this site. Prolonged mortality in the digitate colonies may have been the result of negative growth or partial mortality, which occurred in all the bleached digitate colonies there. The physiological processes impaired by bleaching caused whole or partial coral colony mortality [6,59–61]. Susceptibility to bioerosion may increase in partially dead colonies [62]. Colonization of turf algae on the dead skeletons in partially dead coral colonies may increase microbial activity and degrade the local environment [63].

The mass flux rates determined by colony morphology might explain the different responses to thermal exposure among the various types of *Acropora* corals in this study. Digitate and corymbose colonies had decreased growth rates in t2 at both Sesoko Station and West Sesoko, while that of tabular colonies decreased only at Sesoko Station. Furthermore, extended mortality was observed only for digitate colonies, indicating that digitate and tabular colonies were the most susceptible and resistant to thermal stress, respectively. Some studies have suggested that fast-growing corals with high metabolic rates are relatively more sensitive to thermal anomalies because they accumulate harmful bleaching by-products such as reactive oxygen species (ROS) [10,11,24–26,64]. Growth was slower in digitate colonies than in corymbose and tabular colonies; however, bleaching susceptibility was the greatest in digitate colonies. In contrast to the fast growth hypothesis, mass transfer coefficients of various geometric shapes based on Reynolds-Sherwood numbers calculated by Patterson [65], may corroborate the order of thermal sensitivity in aquatic invertebrates. The relative differences in thermal susceptibility among the colony morphologies observed in the present study followed Patterson’s mass transfer theory (Table 7), with the highest mass flux rates for flat shapes like tabular morphology. Furthermore, digitate colonies had the lowest VRs although they did not significantly differ from those for tabular colonies. The presence of such a pattern, albeit weak, suggests that morphological traits of corals may be associated with enhanced mass flux in branched corals [45].

Size-specific growth decline in response to size-specific mass flux rates may decrease mean corymbose and digitate colony size due to climate change. After the bleaching event investigated in the present study, the growth of larger corymbose and digitate colonies decreased more than it did for the smaller ones. Partial mortality is more likely to occur in larger colonies at both normal [66,67] and high [58,68,69] temperatures. Several studies have shown that smaller colonies were comparatively less affected by high temperature exposure than larger colonies of the same species [20,70,71]. These observations were ascribed principally to the more effective removal of harmful metabolites like ROS because of the relatively higher mass flux in small, flat coral colonies [20,28]. Edmunds and Burges [72] empirically determined that high temperature has more severe negative effects on photosynthesis and respiration in larger whole branching *Pocillopora verrucosa* than it does on smaller ones. These physiological responses, therefore, could also influence coral growth. The size-nonspecific responses of tabular colonies to thermal anomalies observed in the present study may be explained by the fact that tabular colony branch height increases only slightly as the colonies grow. In contrast, height significantly increases as corymbose and digitate colonies grow. Coral colonies with large height to diameter (aspect) ratios have comparatively lower mass flux rates [28], therefore, increasing the aspect ratio with the growth of corymbose and digitate morphologies might explain the observed size-specific thermal responses.

Corals at South Sesoko may have escaped thermal stress or quickly recovered using efficient mass transfer. The growth of larger corymbose and digitate colonies also decreased at South Sesoko. Nevertheless, this site was not as severely affected by thermal anomalies as the other sites. Moreover, this site experienced high partial mortality (Figs 4 and 6) and mortality rates even in t1, suggesting that factors other than temperature were involved here. At all sites, number tags were attached by cable ties to iron rods and used to mark fixed plots. These loosened only at South Sesoko, suggesting that the water movement was strongest at this site. Field studies after the 1998 mass bleaching event reported relatively milder bleaching effect at sites with high water flow [20,73]. High water flow may mitigate coral bleaching by lowering oxidative stress through efficient mass flux [30,74]. Experimental studies also validated that mass transfer in branched corals was higher under oscillatory flow (wave action) than it was under unidirectional flow [45]. This hypothesis should be tested by quantifying water movement in future studies.

High turbidity and increased heterotrophy during and after thermal exposure may have contributed to thermal resistance at Hamamoto and Yakkai. Corals were not thermally stressed at this turbid site. High turbidity alleviates the thermal and solar irradiance effect [75–77] possibly by reducing solar irradiance. In addition, heterotrophic plasticity in some species might acclimatize them to turbid environments by increasing their feeding rate [78]. Increased feeding rate in thermally stressed corals [79] coupled with high organic and nutrient load in turbid environments would lead to higher lipid content enabling corals to maintain their growth and survival rates following a bleaching event [80]. It is possible that *Acropora* at Hamamoto and Yakkai were not thermally stressed due to a combination of the above factors.

### Conclusion

Over the past decades, trait-based approaches, i.e., studying traits of corals such as growth forms, colony size, and growth rate, among coral genera or higher taxa have gained recognition in coral reef ecology [81]. A recent metanalysis showed coral morphology to be a reliable predictor of bleaching variability [82]. However, studies examining intrageneric variability are limited, and the thermal response within the genus *Acropora* is usually inconsistent across studies. For example, digitate *Acropora* were the most thermally sensitive in some studies [26,83], but tabular *Acropora* were found to be more sensitive in other studies [14,84,85]. Such inconstancy might suggest that the local environment, traits of the species studied, or some other factors had roles in governing intrageneric variability. Therefore, it is important to conduct studies of coral bleaching across different environments and temperatures to delineate the roles of morphology, environment, and species-level traits in intrageneric thermal response variability.

In conclusion, our first hypothesis, that bleaching prevalence is driven primarily by thermal exposure, was supported in the present study. Our second hypothesis, that demographic rates recover to normal levels after bleaching was, however, not. Our third hypothesis, that morphological traits of colonies explain differences in thermal exposure response, was also accepted as indicated by the size specific thermal response and morphological thermal hierarchy observed in this study. Overall, future studies investigating the relationships between multiple morphological traits, quantified environmental conditions, and demographic rates can be informative regarding how coral reefs of Sesoko Island, Japan will respond to future climate change.

## Supporting information

S1 Fig Photographs of *Acropora* morphology groups used in present study. (TIF)

S2 Fig Spatio-temporal variation in temperature around Sesoko Island. (TIF)

S3 Fig Cleveland dot charts of growth grouped by sites for all morphologies. Dots highlighted in magenta were the outliers. Outlier colonies were removed from the analysis. One colony has two points (in t1 and t2), both of which were removed, even if only one point was an outlier. In the tabular morphology dot-chart, green and magenta dots are growth of the same colony in t1 and t2, respectively.
(TIF)

S4 Fig Residual plots of digitate morphology. Dashed arrows indicate residual spread patterns.
(TIF)

S5 Fig Residual plots of corymbose morphology. Dashed arrows indicate residual spread patterns.
(TIF)

S6 Fig Residual plots of tabular morphology. Dashed arrows indicate residual spread patterns.
(TIF)

S1 File Nutrient concentration at the mouths of the two rivers near the study sites. (DOCX)

S2 File Supplementary tables cited in text. (DOCX)

S3 File Statistical tests and results of temperature data shown in S2 Fig. (DOCX)

S4 File Data used in this study. (XLSX)
