# Supplementary figures and images for: Effects of moderate thermal anomalies on Acropora corals around Sesoko Island, Okinawa
Source: PLoS One. 2019 Jan 30;14(1):e0210795. doi: 10.1371/journal.pone.0210795 (PMC6353167; doi:10.1371/journal.pone.0210795)

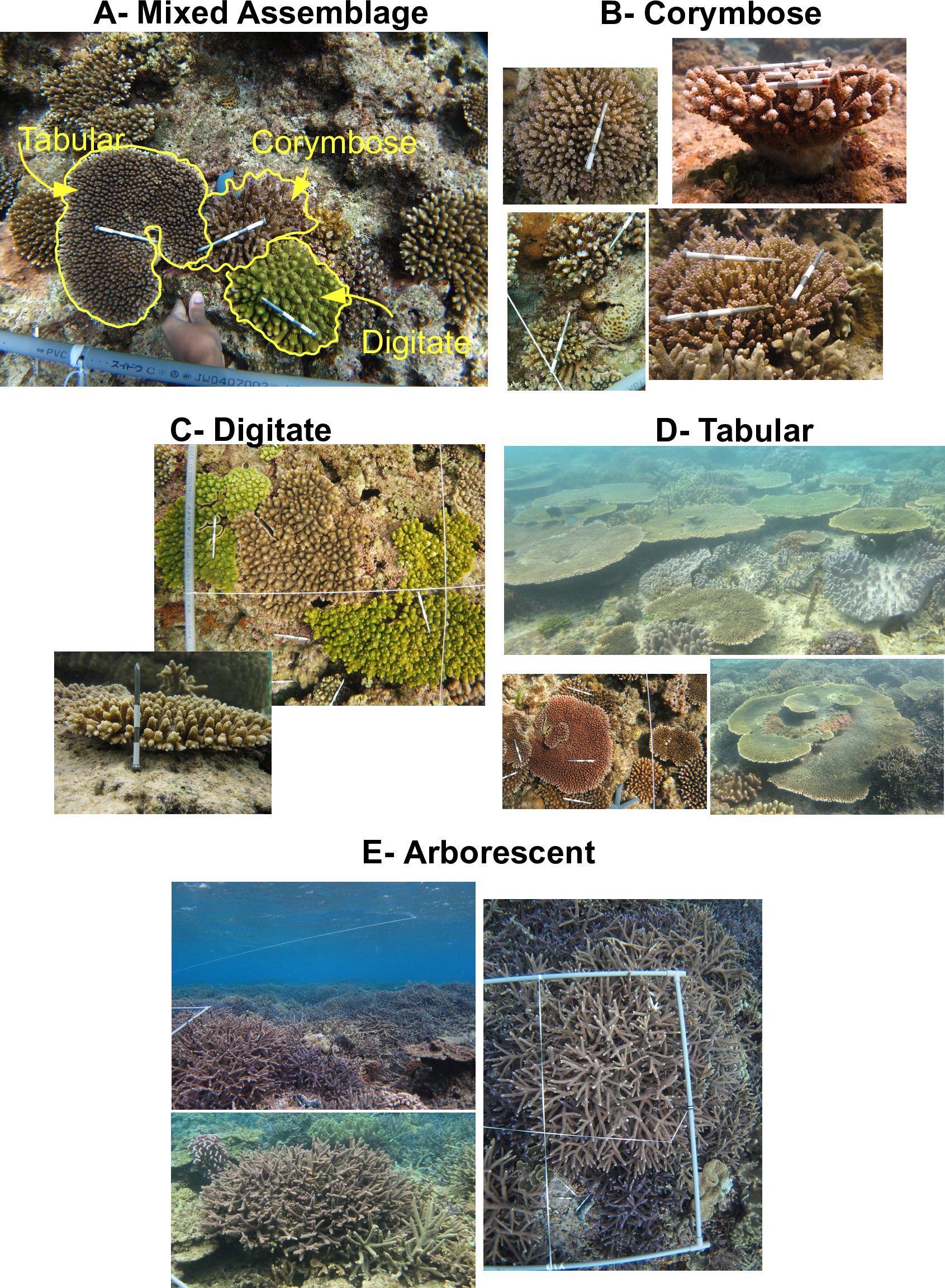

Supplement: S1 Fig — (TIF) [file pone.0210795.s001.tif]

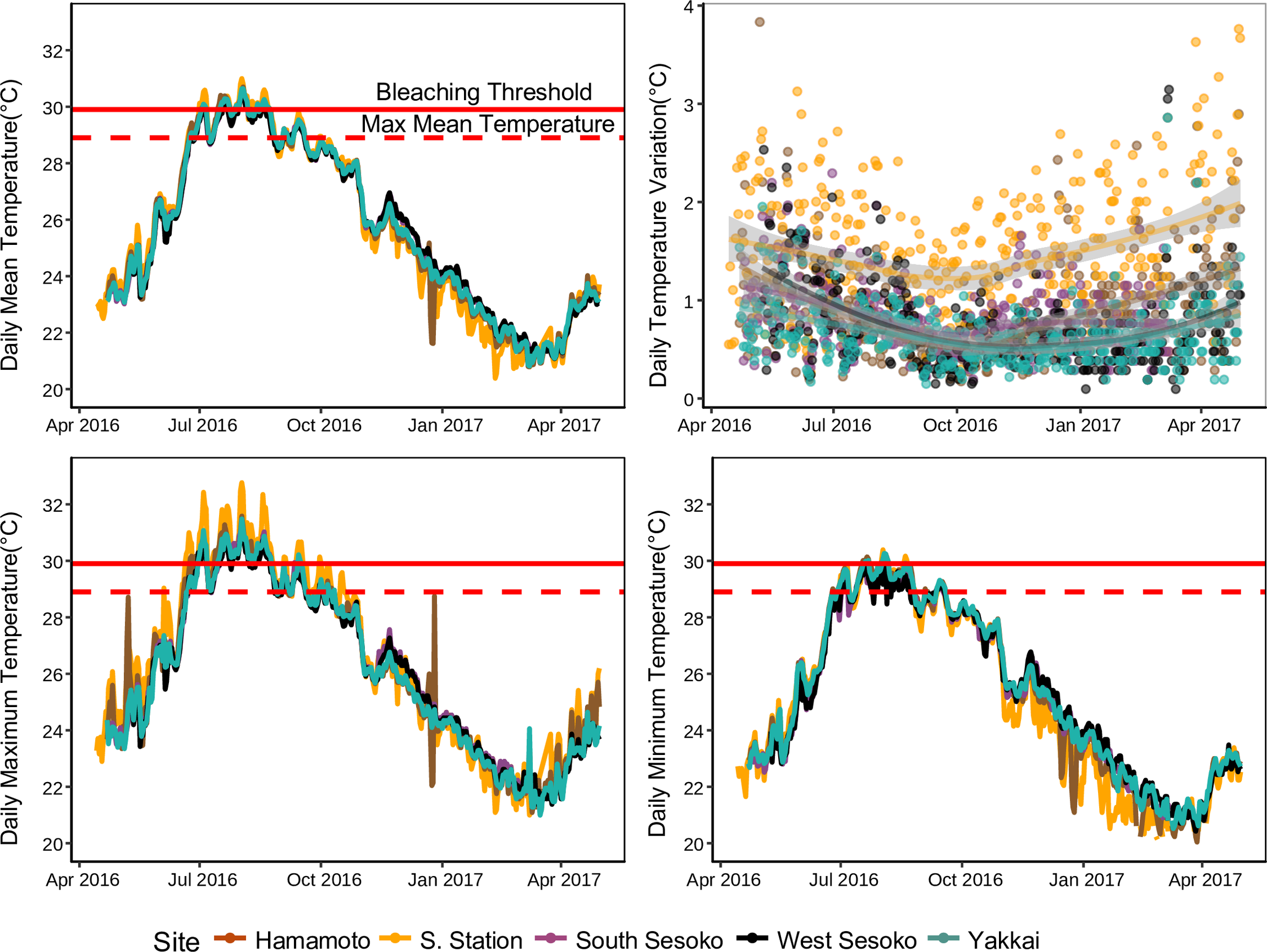

Supplement: S2 Fig — (TIF) [file pone.0210795.s002.tif]

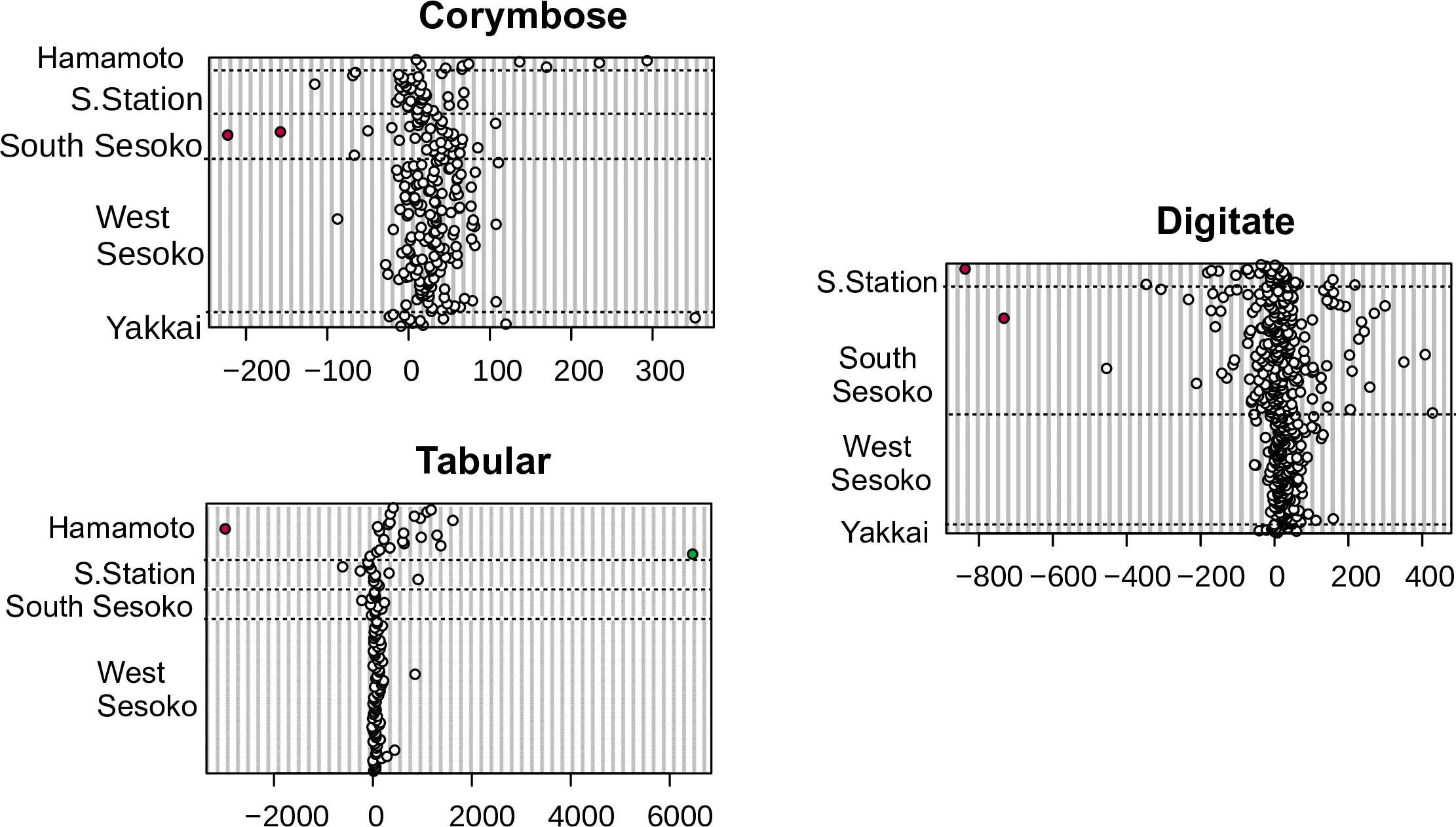

Supplement: S3 Fig — Dots highlighted in magenta were the outliers. Outlier colonies were removed from the analysis. One colony has two points (in t1 and t2), both of which were removed, even if only one point was an outlier. In the tabular morphology dot-chart, green and magenta dots are growth of the same colony in t1 and t2, respectively. (TIF) [file pone.0210795.s003.tif]

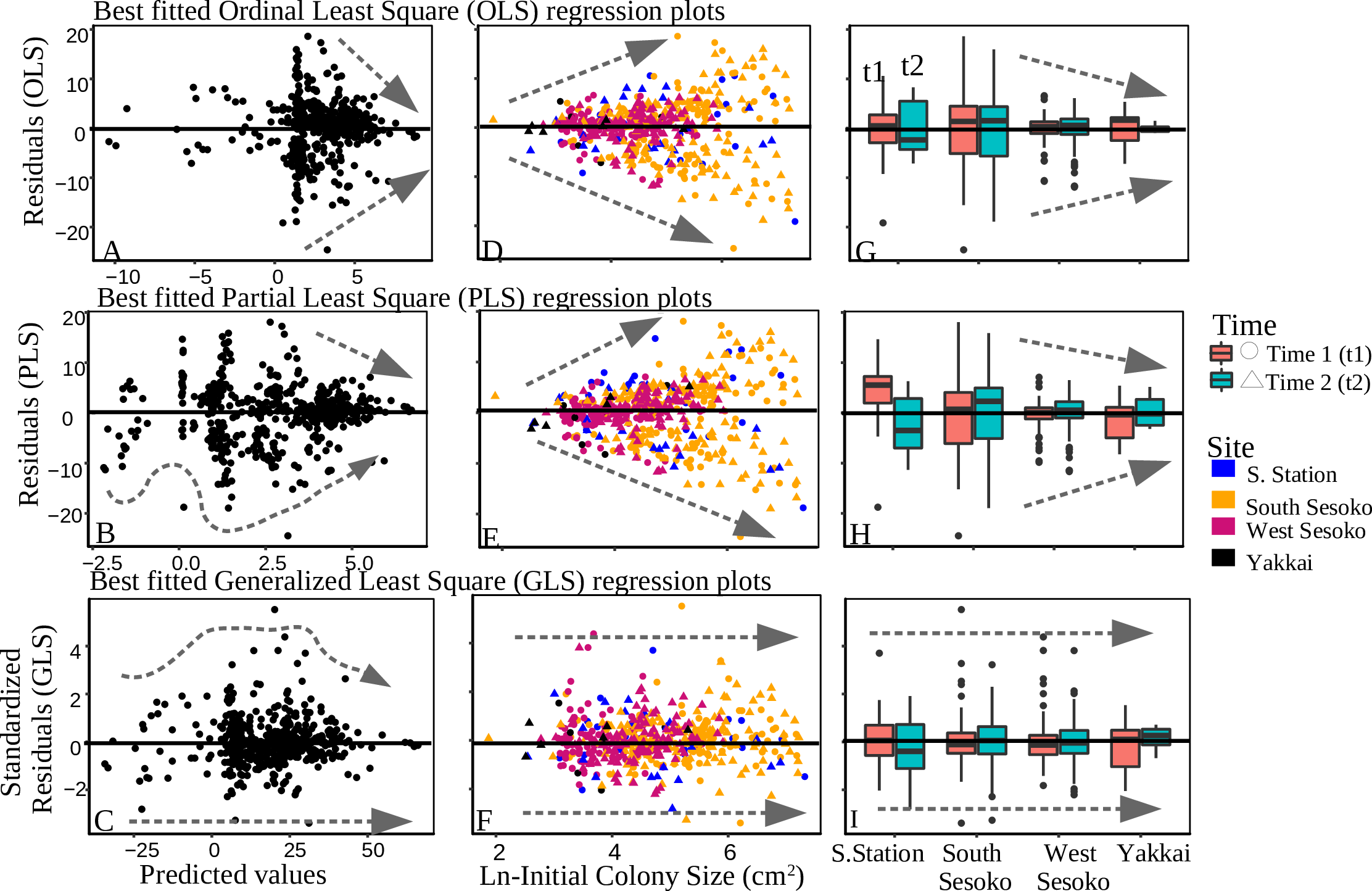

Supplement: S4 Fig — Dashed arrows indicate residual spread patterns. (TIF) [file pone.0210795.s004.tif]

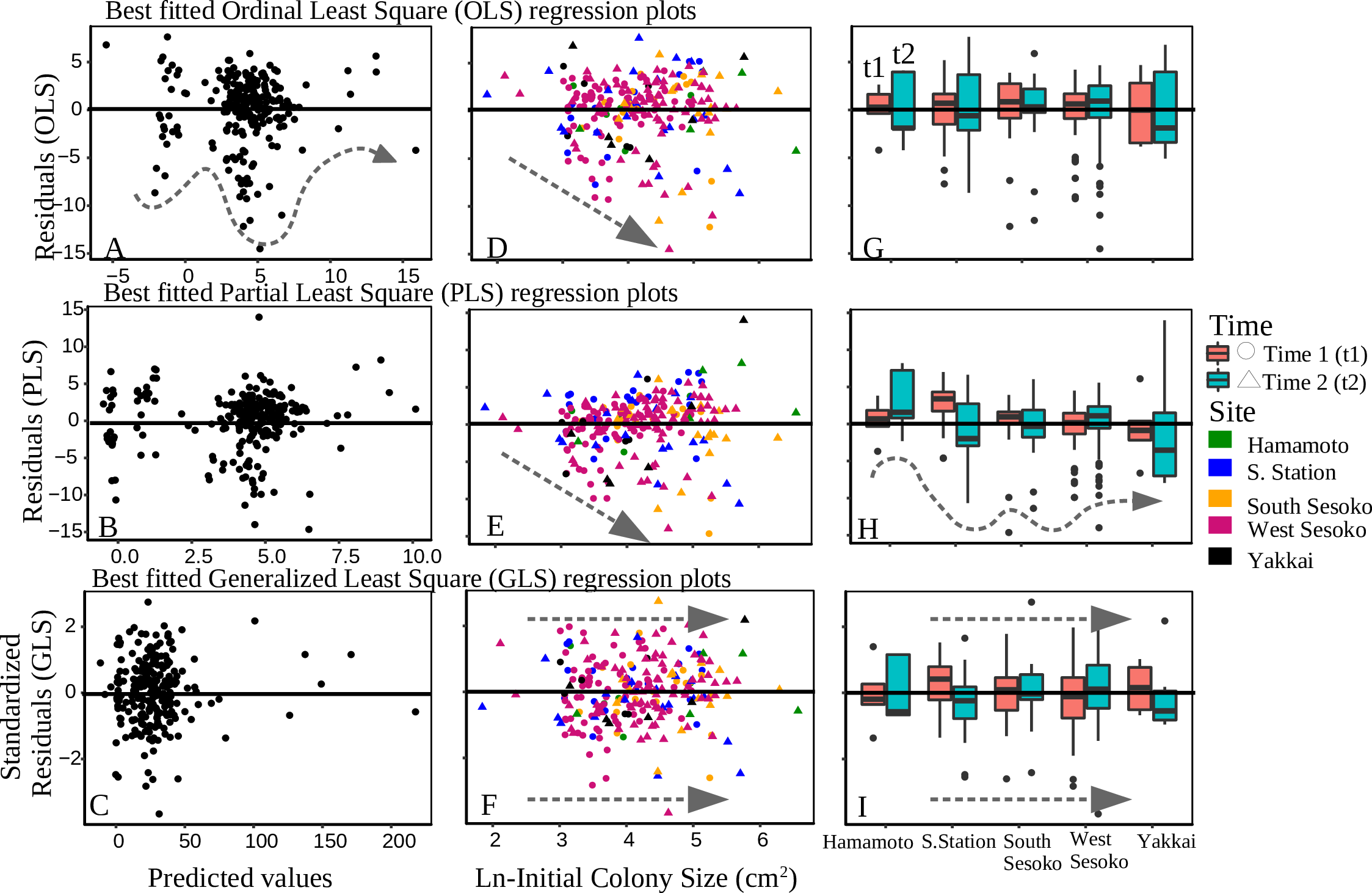

Supplement: S5 Fig — Dashed arrows indicate residual spread patterns. (TIF) [file pone.0210795.s005.tif]

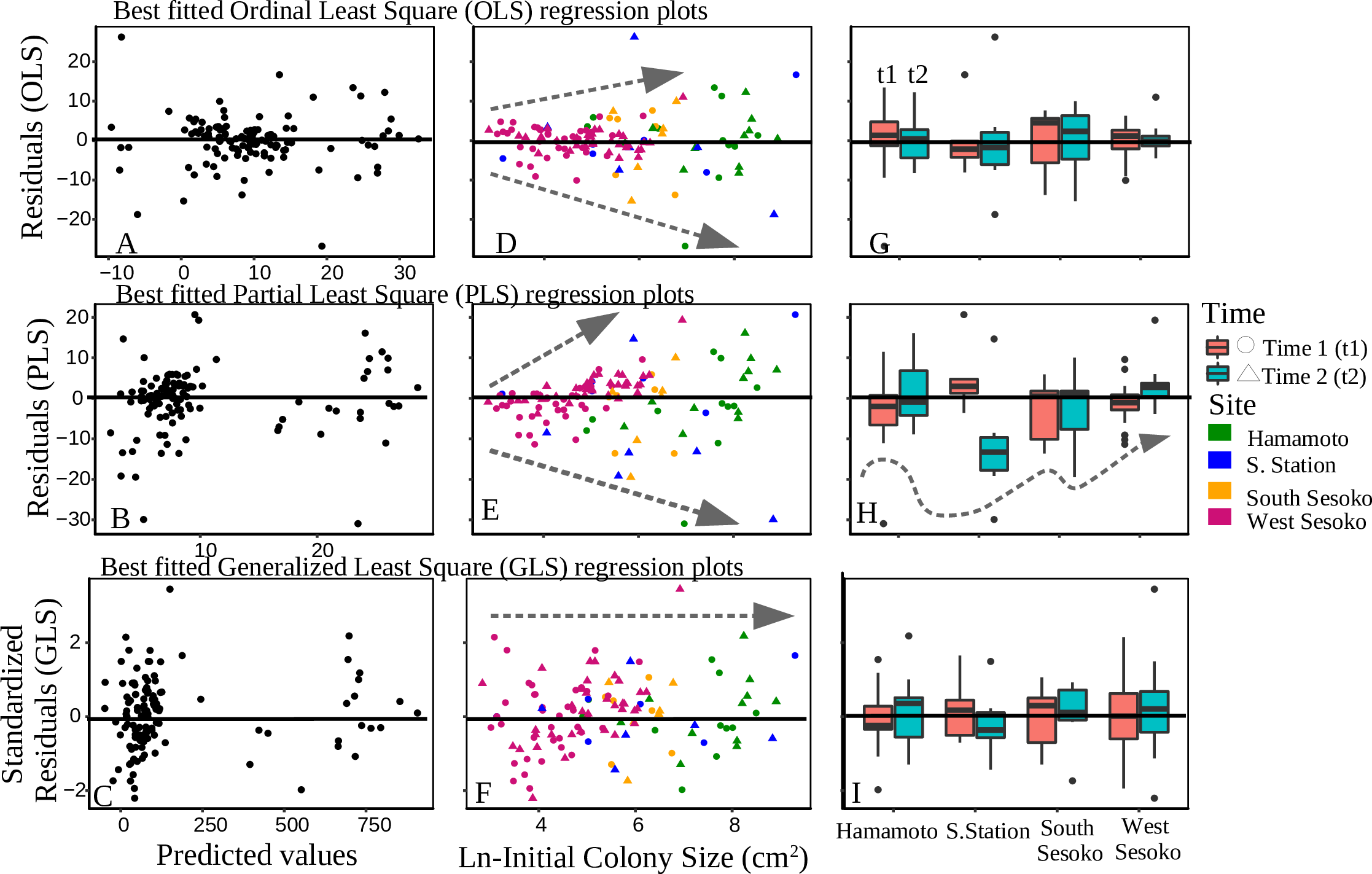

Supplement: S6 Fig — Dashed arrows indicate residual spread patterns. (TIF) [file pone.0210795.s006.tif]
