# Supplementary material for: Effects of moderate thermal anomalies on Acropora corals around Sesoko Island, Okinawa
Source: PLoS One. 2019 Jan 30;14(1):e0210795. doi: 10.1371/journal.pone.0210795 (PMC6353167; doi:10.1371/journal.pone.0210795)
Supplement: S1 File — (DOCX) [file pone.0210795.s007.docx]

**S1 File. Nutrient concentration at the mouths of the two rivers near the study sites.**

Inorganic nutrient concentration was measured twice in June and September 2017 from the two rivers present in the study area (Fig 1). Approximately 100 ml of duplicate samples were collected from the river mouth (0 m or sea surface) at each time point. Ammonium, nitrate, nitrite, phosphate and total phosphate were measured using QuAAtro2-HR auto analyzer (BLTEC Co., Ltd.). Ammonium was measured by indophenol method, nitrate by azo dye formation with cadmium reduction, and phosphate by molybdenum blue method. Both time points in June and one in September (11^th^ September) was following heavy rainfall, when the rivers become highly turbid

| **Nutrient** | **Date** | **River 1(Manna)** | **River 2 (Okobori)** |
| --- | --- | --- | --- |
| Ammonium (µM) | 2017.06.26 | 0 | 0 |
|  | 2017.06.30 | 0.47 ± 0.66 | 0 |
|  | 2017.09.08 | 0 | 0 |
|  | 2017.09.11 | 0.09 ± 0.13 | 0 |
| Nitrite (µM) | 2017.06.26 | 0 | 0 |
|  | 2017.06.30 | 0.07 ± 0.10 | 0 |
|  | 2017.09.08 | 0 | 0 |
|  | 2017.09.11 | 0.1 ± 0.08 | 0 |
| Nitrate (µM) | 2017.06.26 | 0.71 ± 0.09 | 56.37 ± 1.66 |
|  | 2017.06.30 | 70.73 ± 27.93 | 32.13 ± 5.54 |
|  | 2017.09.08 | 1.27 ± 0.64 | 2.84 ± 1.8 |
|  | 2017.09.11 | 29.38 ± 5.81 | 13.69 ± 2.52 |
| Phosphate (µM) | 2017.06.26 | 0.00 | 0.23 ± 0.15 |
|  | 2017.06.30 | 0.83 ± 0.23 | 1.13 ± 0 |
|  | 2017.09.08 | 0.00 | 0.00 |
|  | 2017.09.11 | 0.03 ± 0.04 | 0.41 ± 0.27 |
| Total Phosphate (µM) | 2017.06.26 | 0 | 0 |
|  | 2017.06.30 | 0 | 0 |
|  | 2017.09.08 | 0 | 0 |
|  | 2017.09.11 | 0 | 0 |

**Table1.** Nutrient concentration (mean ± sd ) of two river mouths.
