# Supplementary material for: Effects of moderate thermal anomalies on Acropora corals around Sesoko Island, Okinawa
Source: PLoS One. 2019 Jan 30;14(1):e0210795. doi: 10.1371/journal.pone.0210795 (PMC6353167; doi:10.1371/journal.pone.0210795)
Supplement: S2 File — (DOCX) [file pone.0210795.s008.docx]

**S2 File. Supplementary tables cited in text.**

**Table S1**. Parameter summaries of best-fit GLS models of growth variation for each morphology

| **Morphology** | **Coefficient** | **Value** | **Std. Error** | **t-value** | **p value** |
| --- | --- | --- | --- | --- | --- |
| **Digitate** | (Intercept) | 0.40 | 19.57 | *0.02* | *0.984* |
|  | Time-t2 | 9.19 | 14.97 | *0.61* | *0.531* |
|  | Site-South Sesoko | -36.88 | 17.12 | *-2.15* | *0.032* |
|  | Site-West Sesoko | -61.07 | 18.42 | *-3.31* | *0.001* |
|  | Site-Yakkai | -66.75 | 30.60 | *-2.18* | *0.021* |
|  | Ln-Initial Size | 6.29 | 5.19 | *1.21* | *0.226* |
|  | Time-t2:Site-South Sesoko | 44.13 | 8.63 | *5.11* | *<0.001* |
|  | Time-t2:Site-West Sesoko | 29.94 | 7.68 | *3.90* | *0.0001* |
|  | Time-t2:Site-Yakkai | 35.70 | 11.33 | *3.15* | *0.002* |
|  | **Ln- Initial size:Time-t2** | **-12.65** | **3.76** | ***-3.37*** | ***0.001*** |
|  | Site-South Sesoko:Ln-Initial Size | 4.60 | 4.25 | *1.08* | *0.279* |
|  | Site-West Sesoko:Ln-Initial Size | 16.55 | 4.87 | *3.40* | *0.001* |
|  | Site-Yakkai:Ln-Initial Size | 12.73 | 8.63 | *1.48* | *0.141* |
| **Corymbose** | (Intercept) | -193.98 | 81.32 | *-2.39* | *0.019* |
|  | Site-S.Station | 161.77 | 81.07 | *2.00* | *0.047* |
|  | Site-South Sesoko | 203.48 | 87.78 | *2.32* | *0.021* |
|  | Site-West Sesoko | 129.47 | 81.02 | *1.60* | *0.111* |
|  | Site-Yakkai | 24.36 | 113.60 | *0.21* | *0.830* |
|  | Ln-Initial Size | 69.21 | 21.56 | *3.21* | *0.002* |
|  | Time-t2 | 39.73 | 15.80 | *2.51* | *0.013* |
|  | Site-S.Station:Ln-Initial Size | -58.00 | 21.50 | *-2.70* | *0.008* |
|  | Site-South Sesoko:Ln-Initial Size | -62.44 | 22.81 | *-2.74* | *0.007* |
|  | Site-West Sesoko:Ln-Initial Size | -44.52 | 21.46 | *-2.07* | *0.039* |
|  | Site-Yakkai:Ln-Initial Size | -16.65 | 31.22 | *-0.53* | *0.594* |
|  | **Ln-Initial Size:Time-t2** | **-12.57** | **4.38** | ***-2.87*** | ***0.005*** |
| **Tabular** | (Intercept) | -1063.05 | 244.95 | *-4.34* | *<0.001* |
|  | Site-S.Station | 995.64 | 247.40 | *4.02* | *<0.001* |
|  | Site-South Sesoko | 746.32 | 435.22 | *1.71* | *0.089* |
|  | Site-West Sesoko | 969.74 | 245.47 | *3.95* | *<0.001* |
|  | Ln-Initial Size | 231.91 | 45.49 | *5.10* | *<0.001* |
|  | Time-t2 | -149.93 | 94.78 | *-1.58* | *0.117* |
|  | Site-S.Station :Ln-Initial Size | -204.47 | 46.73 | *-4.38* | *<0.001* |
|  | Site-South Sesoko :Ln-Initial Size | -169.46 | 77.99 | *-2.17* | *0.032* |
|  | Site-West Sesoko :Ln-Initial Size | -196.53 | 45.71 | *-4.30* | *<0.001* |
|  | Site-S.Station :Time-t2 | 57.07 | 104.65 | *0.55* | *0.587* |
|  | Site-South Sesoko :Time-t2 | 78.51 | 113.54 | *0.69* | *0.491* |
|  | Site-West Sesoko :Time-t2 | 148.62 | 94.95 | *1.57* | *0.121* |

**Table S2. AIC values of binomial GLM models of mortality rates at Sesoko Station for each time (t1, tbl, t2).** Best -fit models with lowest AIC values for each time are highlighted. “*” Denotes interaction between two variables.

| S. no. | Model Parameters | Before Bleaching**(t1)** | 1 mo after Bleaching **(tbl)** | 6 mo after bleaching **(t2)** |
| --- | --- | --- | --- | --- |
| 1 | Null Model (none) | **21.44** | **119.73** | 73.40 |
| 2 | Morphology | 23.47 | 122.93 | 63.90 |
| 3 | log-Initital size | 23.44 | 121.73 | 72.63 |
| 4 | log-Initial size + Morph | 25.43 | 124.78 | **63.61** |
| 5 | log-Initial size + Morph + log-Initial Size * Morphology | 29.43 | 125.02 | 67.53 |

**Table S3.** Analysis of deviance table for best-fit binomial GLM model of mortality rates at Sesoko Station in time t2 (6 mo after bleaching).

| Parameter | DF | **Chisq** | **Pr (>Chisq)** |
| --- | --- | --- | --- |
| Ln-Initial Size | 1 | *2.13* | *0.145* |
| Morphology | 2 | *6.12* | ***0.047*** |

**Table S4. Analysis of variance table for void ratio.**

| **Variable** | **Df** | **Sum Sq** | **Mean** | ***F value*** | ***Pr(>F)*** |
| --- | --- | --- | --- | --- | --- |
| Morphology | 2 | 0.50478 | 0.25239 | *8.71* | *0.004* |
| Residuals | 14 | 0.40572 | 0.02898 |  |  |

**Table S5. Post-hoc tests on morphological variation in void ratio**

| **Morphology Pairs** | **Estimate** | **SE** | **df** | **t.ratio** | ***p value*** |
| --- | --- | --- | --- | --- | --- |
| Digitate v/s Corymbose | 0.41 | 0.10 | 14 | *3.97* | ***0.004*** |
| Corymbose v/s Tabular | 0.16 | 0.12 | 14 | *1.29* | *0.425* |
| Tabular v/s Corymbose | -0.25 | 0.10 | 14 | *-2.45* | *0.068* |
| *n* = *9*/digitate;*4*/Corymbose; *4*/Tabular | | | | | |

**Table S6.** Results of best-fit binomial GLM model of mortality rates at Sesoko Station in time t2 (6 months after bleaching).

| Coefficient | Estimate | Std. Error | *z value* | *Pr(>\|z\|)* |
| --- | --- | --- | --- | --- |
| Intercept | -1.47 | 1.42 | *-1.04* | *0.300* |
| Ln-Initial Colony Size | -0.37 | 0.25 | *-1.46* | *0.145* |
| Morphology-Digitate | 2.71 | 1.09 | *2.47* | ***0.013*** |
| Morphology-Tabulate | -13.87 | 1563.69 | *-0.01* | *0.993* |

**Table S7**. Estimates of variables used as variance structure in best-fit GLS models of growth in different morphologies.

| **Morphology** | **Variable** | **Variance-Estimate** |
| --- | --- | --- |
| Digitate | Ln-Initial Size | 3.74 |
|  | Time 1 (t1) | 1 |
|  | Time 2 (t2) | 0.70 |
| Corymbose | Ln-Initial Size | 2.16 |
|  | Hamamoto | 1.00 |
|  | S.Station | 0.45 |
|  | South Sesoko | 0.49 |
|  | West Sesoko | 0.49 |
|  | Yakkai | 1.08 |
| Tabular | Ln-Initial Size | 4.18 |
|  | Hamamoto-t1 | 1.00 |
|  | S.Station-t1 | 0.43 |
|  | South Sesoko-t1 | 0.50 |
|  | West Sesoko-t1 | 1.23 |
|  | Hamamoto-t2 | 0.68 |
|  | S.Station-t2 | 1.42 |
|  | South Sesoko-t2 | 0.79 |
|  | West Sesoko-t2 | 0.68 |

**Table S8**. Post-Hoc tests for “Initial-Size * Site” term used in best-fit growth models of all morphologies. Least-square means of growth are compared between different site pairs. Results are averaged over levels of Time. 0.05 is used as level of significance. df= degrees of freedom; SE = Standard Error

| **Morphology** | **Ln-Initial Size (cm^2^)** | **Site-Pairs** | **Estimate** | **SE** | **df** | **t.ratio** | **p.value** |
| --- | --- | --- | --- | --- | --- | --- | --- |
| Digitate | 4.73 | S.Station - South Sesoko | -6.97 | 7.40 | 479 | -0.94 | 0.783 |
|  |  | **S.Station - West Sesoko** | -32.25 | 7.62 | 479 | -4.23 | **0.000** |
|  |  | S.Station - Yakkai | -11.37 | 15.38 | 479 | -0.74 | 0.881 |
|  |  | **South Sesoko - West Sesoko** | -25.28 | 4.54 | 479 | -5.57 | **<.0001** |
|  |  | South Sesoko - Yakkai | -4.40 | 14.13 | 479 | -0.31 | 0.990 |
|  |  | West Sesoko - Yakkai | 20.88 | 14.23 | 479 | 1.47 | 0.458 |
| Corymbose | 4.17 | **Hamamoto - S.Station** | 80.11 | 18.47 | 228 | 4.34 | **0.000** |
|  |  | **Hamamoto - South Sesoko** | 56.93 | 18.70 | 228 | 3.05 | **0.022** |
|  |  | **Hamamoto - West Sesoko** | 56.19 | 18.17 | 228 | 3.09 | **0.019** |
|  |  | Hamamoto - Yakkai | 45.09 | 27.00 | 228 | 1.67 | 0.455 |
|  |  | **S.Station - South Sesoko** | -23.18 | 6.42 | 228 | -3.61 | **0.003** |
|  |  | **S.Station - West Sesoko** | -23.92 | 4.67 | 228 | -5.13 | **<.0001** |
|  |  | S.Station - Yakkai | -35.02 | 20.51 | 228 | -1.71 | 0.431 |
|  |  | South Sesoko - West Sesoko | -0.74 | 5.53 | 228 | -0.13 | 1.000 |
|  |  | South Sesoko - Yakkai | -11.84 | 20.71 | 228 | -0.57 | 0.979 |
|  |  | West Sesoko - Yakkai | -11.10 | 20.24 | 228 | -0.55 | 0.982 |
| Tabular | 5.43 | Hamamoto - S.Station | 85.87 | 52.00 | 106 | 1.65 | 0.355 |
|  |  | Hamamoto - South Sesoko | 134.42 | 57.74 | 106 | 2.33 | 0.098 |
|  |  | Hamamoto - West Sesoko | 22.88 | 45.00 | 106 | 0.51 | 0.957 |
|  |  | S.Station - South Sesoko | 48.54 | 46.61 | 106 | 1.04 | 0.726 |
|  |  | S.Station - West Sesoko | -63.00 | 29.38 | 106 | -2.14 | 0.146 |
|  |  | **South Sesoko - West Sesoko** | -111.54 | 38.64 | 106 | -2.89 | **0.024** |

**Table S9**. Post-Hoc tests for “Initial-Size * Time” term used in best-fit growth models of all morphologies. Least-square means of growth are compared between different site pairs. Results are averaged over levels of Site. 0.05 is used as level of significance. df= degrees of freedom; SE = Standard Error

| **Morphology** | **Ln-Initial Size (cm^2^)** | **Pairs** | **Estimate** | **SE** | **df** | **t.ratio** | **p.value** |
| --- | --- | --- | --- | --- | --- | --- | --- |
| Digitate | 4.73 | t1 - t2 | 23.26 | 5.52 | 479 | 3.22 | **<.001** |
| Corymbose | 4.17 | t1 - t2 | 12.70 | 3.95 | 228 | 3.22 | **0.002** |
| Tabular | Ln-Initial Size*Time term was not included in best fit model | | | | | | |
