# Supplementary material for: Effects of moderate thermal anomalies on Acropora corals around Sesoko Island, Okinawa
Source: PLoS One. 2019 Jan 30;14(1):e0210795. doi: 10.1371/journal.pone.0210795 (PMC6353167; doi:10.1371/journal.pone.0210795)
Supplement: S3 File — (DOCX) [file pone.0210795.s009.docx]

S3 File. Statistical test and results on temperature data shown in S2 Fig.

1. **Methods: Statistical tests for temperature data.**
   1. Hourly temperature data was obtained from temperature loggers deployed in field. From this following temperature indices were calculated (S2 Fig).
      1. Daily Mean Temperature.
      2. Daily Maximum Temperature.
      3. Daily Minimum Temperature.
      4. Daily temperature variation (Daily Maximum Temperature – Daily Minimum Temperature).
   2. In daily temperature variations datasets, only 4 datapoints had values > 4 ° C. These were considered as outliers and were removed from the dataset.
   3. Generalized additive mixed models (GAMM) with gamma family of distribution and log link function were made for these indices.
   4. First an optimal fixed effects model was selected by minimizing AIC. For all datasets fixed effects were Site and Time (expressed as number of days). A smoothening function was applied to variable Time.
   5. Temporal autocorrelation structure was specified by corARMA function. Following correlation structure was applied. corARMA (Time (in Days) |Site, p = 1, q=1).
   6. corAR1 (Time|Site) is the autocorrelation structure. Here corARMA indicates that it’s a temporal autocorrelation. `*Time|Site*` shows that temporal autocorrelation is within levels of site and not between i.e. that there is temporal autocorrelation at each site. Time is treated as integer because in corArMA Time should be an integer.
   7. p and q are additional parameters. Their values can range from 0 to 3. In this analysis p and q values were considered as 1 for all models.
   8. ANOVA on all temperature GAMM models was done to see the significance of site and time term. In GAMM re

**Results: Statistical tests for temperature data.**

ANOVA tests on GAMM models showed that, all temperature indices varied significantly with time (Table 1; Time *p = <2e -16*). Spatial variation for daily mean temperature was not significant (Table 1; Site *p = 0.842*). Whereas spatial variation was significant for daily maximum (Table 1; Site *p = 0.001*), daily minimum (Table 1; Site *p = 0.004*) and daily temperature variation (Table 1; Site *p < 2e-16*). From GAMM model results (Table 2) we can see that daily maximum temperature and daily temperature variation at Sesoko Station is significantly higher than all other sites. While daily minimum temperature at Sesoko Station is significantly lower than all other sites.

**Table 2: ANOVA results on all GAMM temperature models. df = degrees of freedom.**

| **Response Variable** | **Terms** | **df** | ***F-value*** | ***p-value*** |
| --- | --- | --- | --- | --- |
| Daily Mean temperature | Site | 4 | *0.35* | *0.842* |
|  | **Time** | 8.79 | *741.90* | ***<2e-16*** |
| Daily Maximum temperature | **Site** | 4 | *4.90* | ***0.001*** |
|  | **Time** | 8.80 | *844.30* | ***<2e-16*** |
| Daily Minimum temperature | **Site** | 4 | *3.86* | ***0.004*** |
|  | **Time** | 8.76 | *629.10* | ***<2e-16*** |
| Daily temperature variation | **Site** | 4 | *66.88* | ***<2e-16*** |
|  | **Time** | 6.29 | *20.75* | ***<2e-16*** |

**Table 2. GAMM model results showing significant spatial variation of different temperature indices.** Interpretation of table: For all indices, site Sesoko Station is the reference term. Therefore, intercept of daily maximum temperature at Hamamoto is 0.01 times lower than that of Sesoko Station. Note: the results (Estimate and Std error) are on log scale.

| **Response variable** | **Parametric coefficients** | **Estimate** | **Std. Error** | **t value** | **Pr(>\|t\|)** |
| --- | --- | --- | --- | --- | --- |
|  |  |  |  |  |  |
| Daily Maximum temperature | (Intercept) | 3.27 | 0.003 | 1187.32 | ***< 2e-16*** |
|  | Hamamoto | -0.01 | 0.004 | -2.74 | ***0.006*** |
|  | South Sesoko | -0.01 | 0.004 | -2.61 | ***0.009*** |
|  | West Sesoko | -0.01 | 0.004 | -3.74 | ***<0.001*** |
|  | Yakkai | -0.02 | 0.004 | -3.90 | ***9.9E-05*** |
| Daily Minimum temperature | (Intercept) | 3.22 | 0.003 | *943.97* | ***< 2e-16*** |
|  | Hamamoto | 0.01 | 0.005 | *2.34* | ***0.02*** |
|  | South Sesoko | 0.01 | 0.005 | *2.90* | ***0.00*** |
|  | West Sesoko | 0.01 | 0.005 | *3.06* | ***0.00*** |
|  | Yakkai | 0.02 | 0.005 | *3.48* | ***0.00*** |
| Daily temperature variation | (Intercept) | 0.38 | 0.038 | *9.94* | ***<2e-16*** |
|  | Hamamoto | -0.54 | 0.054 | *-10.09* | ***<2e-16*** |
|  | South Sesoko | -0.57 | 0.057 | *-10.00* | ***<2e-16*** |
|  | West Sesoko | -0.71 | 0.055 | *-13.01* | ***<2e-16*** |
|  | Yakkai | -0.81 | 0.054 | *-15.00* | ***<2e-16*** |
